# Demand and level of service inflation in Floating Catchment Area (FCA) methods

**DOI:** 10.1371/journal.pone.0218773

**Published:** 2019-06-27

**Authors:** Antonio Paez, Christopher D. Higgins, Salvatore F. Vivona

**Affiliations:** 1 School of Geography and Earth Sciences, McMaster University, 1280 Main St W, Hamilton, ON L8S 4K1 Canada; 2 Department of Land Surveying and Geo-Informatics & Department of Building and Real Estate, 11 Yuk Choi Rd, Hung Hom, Hong Kong; 3 Department of Computer Science, University of Toronto, 214 College Street, Toronto, ON, M5T 3A1 Canada; The University of the South Pacific, FIJI

## Abstract

Floating Catchment Area (FCA) methods are a popular tool to investigate accessibility to public facilities, in particular health care services. FCA approaches are attractive because, unlike other accessibility measures, they take into account the potential for congestion of facilities. This is done by 1) considering the population within the catchment area of a facility to calculate a variable that measures level of service, and then 2) aggregating the level of service by population centers subject to catchment area constraints. In this paper we discuss an effect of FCA approaches, an artifact that we term demand and level of service *inflation*. These artifacts are present in previous implementations of FCA methods. We argue that inflation makes interpretation of estimates of accessibility difficult, which has possible deleterious consequences for decision making. Next, we propose a simple and intuitive approach to proportionally allocate demandand and level of service in FCA calculations. The approach is based on a standardization of the impedance matrix, similar to approaches popular in the spatial statistics and econometrics literature. The result is a more intiuitive measure of accessibility that 1) provides a local version of the provider-to-population ratio; and 2) preserves the level of demand and the level of supply in a system. We illustrate the relevant issues with some examples, and then empirically by means of a case study of accessibility to family physicians in the Hamilton Census Metropolitan Area (CMA), in Ontario, Canada. Results indicate that demand and supply inflation/deflation affect the interpretation of accessibility analysis using existing FCA methods, and that the proposed adjustment can lead to more intuitive results.

## Introduction

An important issue in health geography and health policy is the evaluation of accessibility to healthcare services, with hundreds of research papers published on the topic since the 2000s [1]. However, the concept of accessibility is multi-dimensional, which often presents challenges to its operationalization in empirical research. According to Joseph and Bantock [2], accessibility can be defined by both aspatial and spatial dimensions. The first dimension considers factors such as the quality of the services and their cost, as well as the income, social class, ethnicity, and mobility profile of potential users of services. From a geographical perspective, the spatial dimension is key, and considers the distribution of available healthcare services across the landscape, in addition to the cost or friction that potential users incur when trying to reach these services. By taking these geographical factors into account, estimates of accessibility can help researchers, planners, and policy makers identify areas with high or low accessibility to healthcare services. This, in turn, can provide valuable information related to social and spatial inequalities and guidance for health policy and resource allocation.

Spatial accessibility can be estimated in various ways. At a high level, provider-to-population ratios (PPR) offer an indication of the level of service within a community. These measures conceptualize a region as a container of population and services, and therefore are sometimes called container approaches. PPRs are straightforward to interpret as the supply of a service (say number of doctors, beds, etc.) divided by demand (say, number of people who require the service). Despite this convenient and intuitive interpretation, container approaches are limited in the amount of spatial information that they provide, especially if applied to large regions. When applied to smaller regions these approaches present other shortcomings, such as the assumption that the population in the container is captive and does not cross the boundaries of the container in search of services—and that users do not come into the container from other regions to avail themselves of local services.

An alternative to container approaches is provided by gravity measures. Gravity measures offer a more sophisticated approach to measuring spatial accessibility to healthcare [2] that moreover addresses some of the limitations of the container approach. Instead of defining rigid container boundaries, gravity measures consider the mobility characteristics of the public to produce flexible (and often overlapping) catchment areas for both services and population. Accordingly, one of the most popular approaches for estimating healthcare accessibility in the literature is the Two-Step Floating Catchment Area (2SFCA) method proposed by Luo and Wang [3] after research by Radke and Mu [4]. The 2SFCA method is an ensemble of two gravity models with a simplified binary distance function to account for crowding of facilities and allocation of levels of service. Numerous applications of this methods are found in the international literature, including work from Germany [5], South Korea [6], Japan [7], China [8], Australia [9], and Canada [10].

Accessibility to healthcare is estimated in two stages in the 2SFCA: in the first step, a level of service at a given healthcare provider is determined based on the supply (e.g., number of physicians in a clinic) and the estimated demand from the surrounding population within some catchment area. This level of service resembles a local provider-to-population ratio (PPR). In the second step, the level of service of different healthcare providers is aggregated for each population center. By operationalizing accessibility in terms of demand and level of service, the 2SFCA method is appealing for health policy analysis. Still, several improvements have been proposed that seek to address the method’s most important perceived shortcomings. The result is a family of Floating Catchment Areas (FCA) methods that include more realistic conceptualizations of the friction of distance by specifying variable catchment area sizes [9] and/or the use of stepped [11], continuous [12], and adaptive [13] distance-decay functions. Other authors have added multi-modal transportation [14], age-adjusted healthcare demand profiles [15], as well as ways to counteract the modifiable areal unit problem [16].

A major focus of FCA research, in addition to the improvements mentioned above, has been the introduction of competition for available opportunities or the allocation of services to the population. More concretely, the original 2SFCA approach has been criticized for over-estimating the levels of demand [17] and/or level of service [18] in the system. This is a consequence of the way catchment areas for facilities and population centers typically overlap in any realistic spatial system—an artifact of FCA methods that can lead to misleading estimates of accessibility.

In effect, when aggregating the population within the overlapping catchment areas of multiple facilities, the original 2SFCA framework leads to double-counting of the population that tends to inflate the level of demand at supply points in the healthcare system. We call this effect *demand inflation*. Inflated demand, in turn, tends to *deflate* the level of service for populations serviced by the facilities so affected. A similar effect, which we call *level of service inflation*, happens when the levels of service of various service points are aggregated for population centers. Ultimately, accessibility estimates are affected in potentially complex ways, depending on the geography of the problem [18], and their interpretation as PPRs becomes suspect.

Various solutions to the issues of demand and level of service inflation have been proposed, including the addition of selection weights based on a travel impedance function in the Three-Step Floating Catchment Area (3SFCA) method [17]; the use of a Huff model to generate probability-based estimate of the selection weights in the 3SFCA method [19]; and, on the supply side, a modified 2SFCA (M2SFCA) method to address suboptimal spatial configuration of services [18].

In this paper we are interested in the way demand and level of service are calculated in FCA methods. We review how different approaches deal with the issue of inflation, and then propose a simple and intuitive approach to proportionally allocate supply and demand. Our solution consists on adjusting the impedance weights used in the estimation of FCA methods. More concretely, by incorporating methods drawn from the field of spatial statistics and econometrics, proportional allocation has the feature that it preserves the levels of demand and service in the system. To illustrate the key aspects of our proposal, we conduct a case study of access to family physicians in Hamilton, Canada. Our results indicate that the proposed adjustments produce more intuitive measures of accessibility to healthcare measured in terms of local PPRs. Moreover, these outputs can be used to provide estimates of access disparity across a region that are both easily understood and robust to demand and level of service inflation.

## Background: Floating Catchment Area methods

To motivate the discussion to follow we begin by reviewing some popular FCA methods. In general terms, FCA approaches are implemented as ensembles of two gravity models in two steps, using an impedance function to represent the cost required to overcome distance. Impedance functions implement a distance-decay effect that mimics a commonly observed cost-minimization behavior, namely that people in general prefer to spend less time/money/effort travelling to destinations. In this way, the impedance function defines a *catchment area* for the points of service and population centers alike.

In the first step of FCA methods, the impedance function defines catchment areas for facilities *j*, which could be clinics, parks, libraries, etc. A weighted sum of the population within a catchment area is allocated to the corresponding facility or service point to represent demand. In the second step of the algorithm, the catchment areas are “floated” to population centers *i*. Accessibility at location *i* is calculated as the weighted sum of the level of service at every location *j* that includes *i* within its catchment area. The following methods are popular in the literature.

### Two-Stage Floating Catchment Areas (2SFCA)

The original 2SFCA implements a binary impedance function *W* with a threshold cost *d*_0_ as follows [3]:

$$W\left(d_{ij}\leq d_{0}\right)=\{\begin{array}{cc} 1 & \;d_{ij}\leq d_{0}\\ 0 & \;d_{ij}>d_{0} \end{array}$$

This function assumes equal potential within a catchment area (i.e., *d*_*ij*_ ≤ *d*_0_), and zero beyond (*d*_*ij*_ > *d*_0_). This implies that 1) travellers are equally likely users of a service point within the catchment area, irrespective of how proximate or distant they are from it; and 2) no users travel to the service point from beyond the threshold cost.

Given the impedance function, the level of demand *D*_*j*_ is calculated as the weighted sum of the population at *i*:

$$D_{j}=\sum_{i}D_{ij}=\sum_{i}P_{i}W\left(d_{ij}\leq d_{0}\right)$$

The supply *S* of the service offered at location *j* (say, number of beds/doctors in a clinic) is then divided by the demand to obtain a measure of level of service (e.g., beds/person, sq.m of park space/person, library floor space/person). This gives a level of service *L*_*j*_ at the service point:

$$L_{j}=\frac{S_{j}}{D_{j}}=\frac{S_{j}}{\sum_{i}D_{ij}}=\sum_{i}\frac{S_{j}}{D_{ij}}=\sum_{i}L_{ij}$$

The level of service resembles a PPR. Aggregation of demand creates a congestion effect that depends on the number of potential users from different origins *i* that converge at service point *j*: at a fixed level of supply, greater demand results in lower levels of service. The different decompositions of *L*_*j*_ help to understand how different population centers contribute to the level of demand at facility *j*.

In the second step of the algorithm, catchment areas are “floated” to population centers *i*. A second gravity model is used to calculate the accessibility at *i*:

$$A_{i}=\sum_{j}L_{j}W\left(d_{ij}\leq d_{0}\right)$$

Since accessibility is calculated as the weighted sum of the level of service at facilities, it is conventionally interpreted as a PPR.

### Enhanced Two-Stage Floating Catchment Areas (E2SFCA)

A criticism of the binary impedance function of the 2SFCA is that it does not account for the declining probability of using a facility as distance grows. As a result of this criticism, other impedance functions have since been proposed, including the stepwise formulation of the Enhanced Two-Stage Floating Catchment Area method [11]:

$$W\left(d_{ij}|d_{1},d_{2},\ldots ,d_{R}\right)=\{\begin{array}{cc} k_{1} & \;d_{ij}\leq d_{1}\\ k_{2} & \;d_{1}<d_{ij}\leq d_{2}\\ \ldots \\ k_{R-1} & \;d_{R-1}<d_{ij}\leq d_{R}\\ 0 & \;d_{ij}>d_{R} \end{array}$$

A stepwise function does not assume identical potential within the catchment area (i.e., the space contained within *d*_*ij*_ ≤ *d*_*R*_), but rather declining potential with increasing cost of travel. It is worthwhile noting that impedance functions have long been studied in geographical analysis in general [20], and accessibility research in particular [21]. However, it is only relatively recently that alternative impedance functions have been incorporated in FCA approaches, including continuous functions [12] and mixtures of continuous and step functions [22].

Besides the use of a non-binary impedance function, the method remains the same. In the first step, demand is calculated as a weighted sum of the population within the catchment area:

$$D_{j}=\sum_{i}D_{ij}=\sum_{i}P_{i}W\left(d_{ij}|d_{1},d_{2},\ldots ,d_{R}\right)$$

Note that non-binary impedance functions discount the level of demand as a function of cost more rapidly than binary functions. How rapidly this happens depends on the definition of the cutoff values *d*_1_, *d*_2_, ⋯, *d*_*R*_ and weights *k*_1_, *k*_2_, ⋯, *k*_*r*−1_ of the function.

In the second step of the algorithm, accessibility at *i* is calculated as the weighted sum of the level of service of service points *j*:

$$A_{i}=\sum_{j}\frac{S_{j}}{D_{j}}W\left(d_{ij}|d_{1},d_{2},\ldots ,d_{R}\right)=\sum_{j}L_{j}W\left(d_{ij}|d_{1},d_{2},\ldots ,d_{R}\right)$$

Again, the use of a non-binary impedance function discounts the level of service more rapidly compared to binary functions.

### Three-Stage Floating Catchment Areas (3STCA)

Wan et al. [17] proposed a Three-Stage Floating Catchment Area method (3SFCA) that aims at refining the estimates of level of demand and accessibility by means of the use of *selection weights*. This approach operates by introducing an aditional step where selection weights are calculated as follows:

$$G_{ij}=\frac{T(d_{ij})}{\sum_{j\forall d_{ij}\leq d_{0}}T\left(d_{ij}\right)}$$

where *T*(*d*_*ij*_) are Gaussian weights (essentially an impedance function), and the summation in the denominator is for all sites *j* that are within a critical threshold *d*_0_. Notice that a property of the selection weights is that their sum over *j* equals one:

$$\sum_{j}G_{ij}=1$$

Given a set of selection weights, the level of demand is caclulated by this algorithm in the following manner:

$$D_{j}^{*}=\sum_{i}G_{ij}P_{i}W\left(d_{ij}|d_{1},d_{2},\ldots ,d_{R}\right)=\sum_{i}G_{ij}D_{ij}$$

Notice how demand in this method takes what is essentially the demand in the E2SFCA, and allocates it proportionally to service points *j*.

Accessibility, in the final step, becomes (with the subindices of the selection weights reversed, to reflect the displacement of the catchment area to population centers) is calculated in the following manner:

$$A_{i}^{*}=\sum_{j}G_{ji}\frac{S_{j}}{D_{ij}^{*}}W\left(d_{ij}|d_{1},d_{2},\ldots ,d_{R}\right)=\sum_{j}G_{ji}L_{j}^{*}W\left(d_{ij}|d_{1},d_{2},\ldots ,d_{R}\right)$$

### Modified Two-Stage Floating Catchment Areas (M2SFCA)

Delamater [18] discusses the application of FCA methods for systems that are not optimally configured to service the whole population. To address this issue, he proposes a modification to the second step of the 2SFCA algorithm that increases the friction of distance. Demand in this modification is the same as in 2SFCA. However, accessibility is calculated in the following manner:

$$A_{i}=\sum_{j}L_{j}W\left(d_{ij}|d_{1},d_{2},\ldots ,d_{R}\right)W\left(d_{ij}|d_{1},d_{2},\ldots ,d_{R}\right)=\sum_{j}L_{j}(W(d_{ij}|d_{1},d_{2},\ldots ,d_{R}){)}^{2}$$

In other words, the level of service is discounted by the square of the impedance function, thus increasing the rate of decay. This is done to reflect the possibility that some population centers may experience increased friction to reach destinations in suboptimally configured systems.

## Inflation effects in FCA methods

Having reviewed a selection of FCA approaches, we now proceed to discuss the issue of inflation. Inflation has been identified, among others, by Wan et al. [17] and Delamater [18]. As discussed by these authors, inflation happens when demand or level of service are overestimated. Inflation is a consequence of the way in which *Dj* and *A*_*i*_ are calculated, with some population centers contributing to the level of demand at more than one facility and then the level of service of facilities allocated to multiple population centers. Calculating demand, in particular, generally fails to preserve the population, and therefore lacks the pycnophilactic property discussed by Tobler [23]. In practical terms, this implies that the population used to calculate the demand component of level of service will often exceed (but sometimes fall short of) the actual population in a region, depending on the weighting scheme. We term the consequent effect *demand inflation*.

Let us illustrate this inflation effect by means of a simple example using the conventional 2SFCA approach with a binary impedance function. In this case, the population value at *i* is multiplied by zero or one, meaning that the contribution of *i* to demand at *j* whenever *d*_*ij*_ does not exceed the threshold is:

$$D_{ij}=P_{i}$$

**Fig 1:**
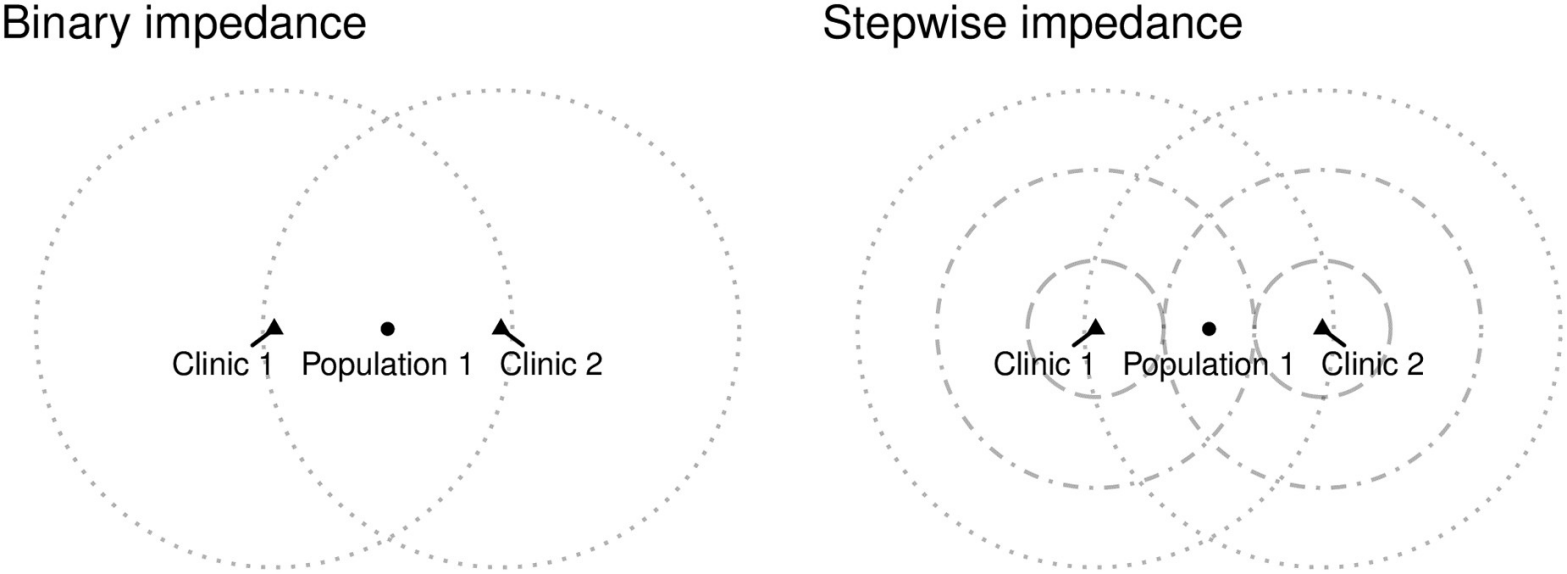
A single population center that enters the service areas of two clinics (triangles are clinics, dotted lines are segments of catchment areas).

If we concentrate for a moment on a single population center that enters the catchment areas of several service points (see Fig 1, left panel), we can see that when the demand at each of the service points is calculated, the population in question is added two times, and the levels of service are *L*_1_ = *L*_2_ = 1/*s*00.

More generally, when calculating the level of service at *L*_*j*_, the population at *i* contributes to demand every time that *d*_*ij*_ ≤ *d*_0_ for any *j*. And, since since *D*_*i*_ *j* = *P*_*i*_, it follows that the sum of the population to be serviced over all clinics is:

$$\sum_{j}D_{ij}=K_{i}P_{i}$$

where *K*_*i*_ is the number of service points *j* that include *i* as part of their catchment areas. Therefore, the system-wide contribution of the population at *i* to the level of demand implied by these calculations, vastly exceeds the actual population at *i*, since:

$$\sum_{j}D_{ij}=K_{i}P_{i}>P_{i}$$

Let us consider next what happens when enhanced (i.e., non-binary) impedance weights are used. These functions aim to capture more realistically the rule that most members of the population prefer to travel shorter distances to reach a destination. For the example, assume a set of weights with decay as follows (see Fig 1, right panel):

$$W\left(d_{ij}|d_{1},d_{2},\ldots ,d_{R}\right)=\{\begin{array}{cc} 0.9 & \;d_{ij}\leq d_{1}\\ 0.8 & \;d_{1}<d_{ij}\leq d_{2}\\ 0.4 & \;d_{R-1}<d_{ij}\leq d_{R}\\ 0 & \;d_{ij}>d_{R} \end{array}$$

The population center in the example is relatively distant from the service points. Accordingly, its potential demand is reduced by assuming that some people do not travel at all. In this example, the contribution of the population center to demand is only 0.8*P* to each clinic, and therefore the system-wide demand of this center is 1.6*P*—less than the all-or-nothing allocation of the binary impedance weights, but still in excess of the actual population.

More generally, when calculating the level of service at *j* locations, the population at *i* contributes to demand every time that *d*_*ij*_ is within the service area for any *j*. The precise contribution depends on the weights in the distance-decay function and the position of the population center with relative to all service points. In a function with faster decay, the total demand attributed to *i* (i.e., ∑_*i*_ *D*_*ij*_) can be less than the population of *i*. In other words, depending on the steepness of decay, the total demand can be greater than, equal to, or less than the population at *i*:

$$\sum_{j}D_{ij}≶P_{i}$$

Clearly, only when the full population at *i* is allocated exclusively to one service point (i.e., when *K*_*i*_ = 1) the implied demand equals the population—something that seldom happens in practical situations.

It is important to acknowledge that demand in accessibility analysis represents the *potential* for spatial interaction, not realized interaction. That said, the expectation that facilities need to serve multiple times the size of the population in a region can easily lead to misleading conclusions about the need for resources. A logical question, however, is whether the inflation of demand (with the consequence deflation of level of service) is not offset in the second step of the method, when the population at *i* has potential access to multiple service points?

**Fig 2:**
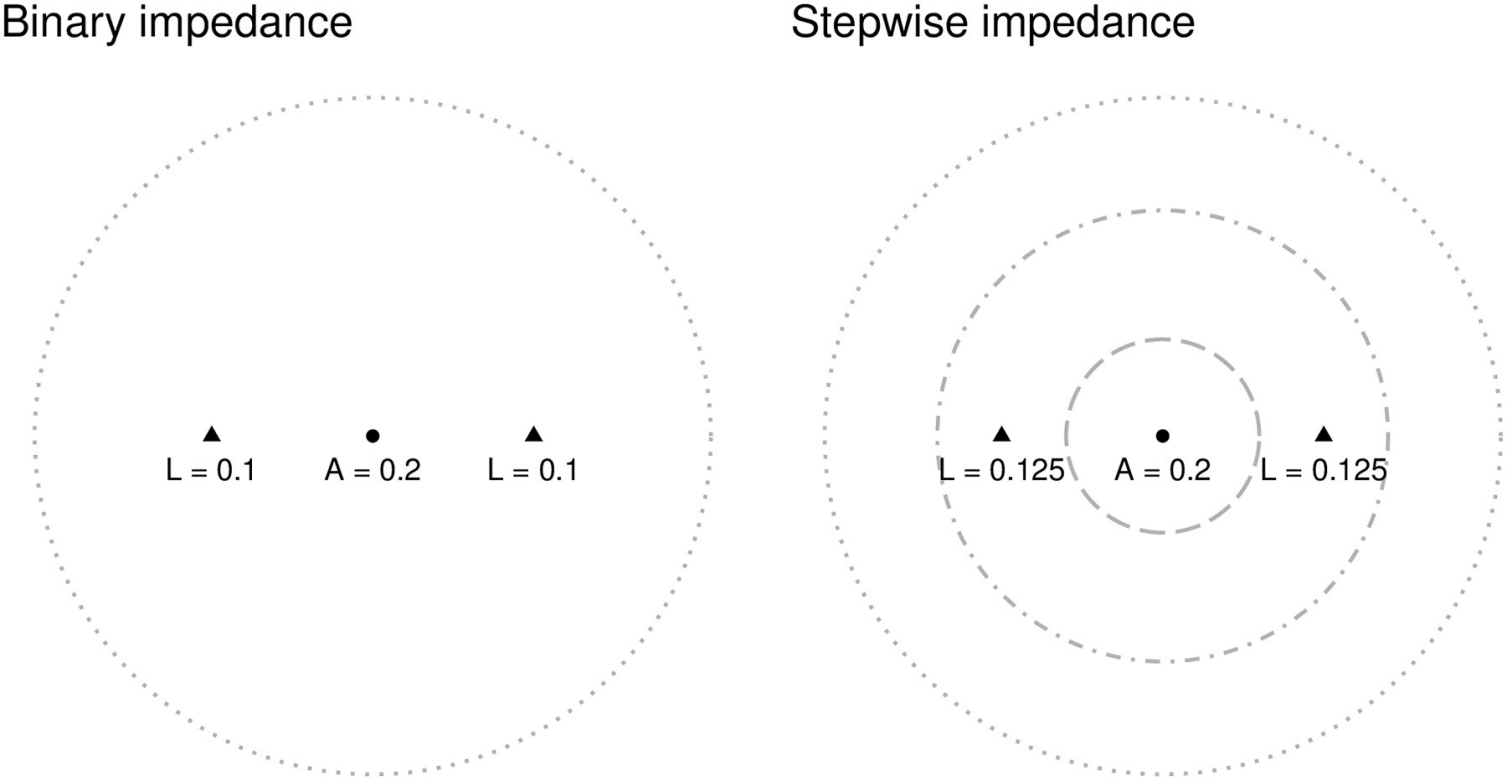
Levels of service of three clinics and accessibility of one population center (triangles are clinics, dotted lines are segments of catchment areas).

Let us consider what happens in the second step of the algorithm in the example, when catchment areas are floated to the population center (see Fig 2). When a binary impedance function is used, the aggregation of the level of service means that, despite the inflation of demand due to double-counting, accessibility matches the level of service *as well as* the regional PPR of 2/100 (left panel). In the case of the stepwise function, the level of implied demand is less than the population, but the population is also assumed to receive less of the available level of service. In this case, again, the accessibility matches the level of service *despite the fact that segments of the population were assumed to not contribute to demand*.

**Fig 3:**
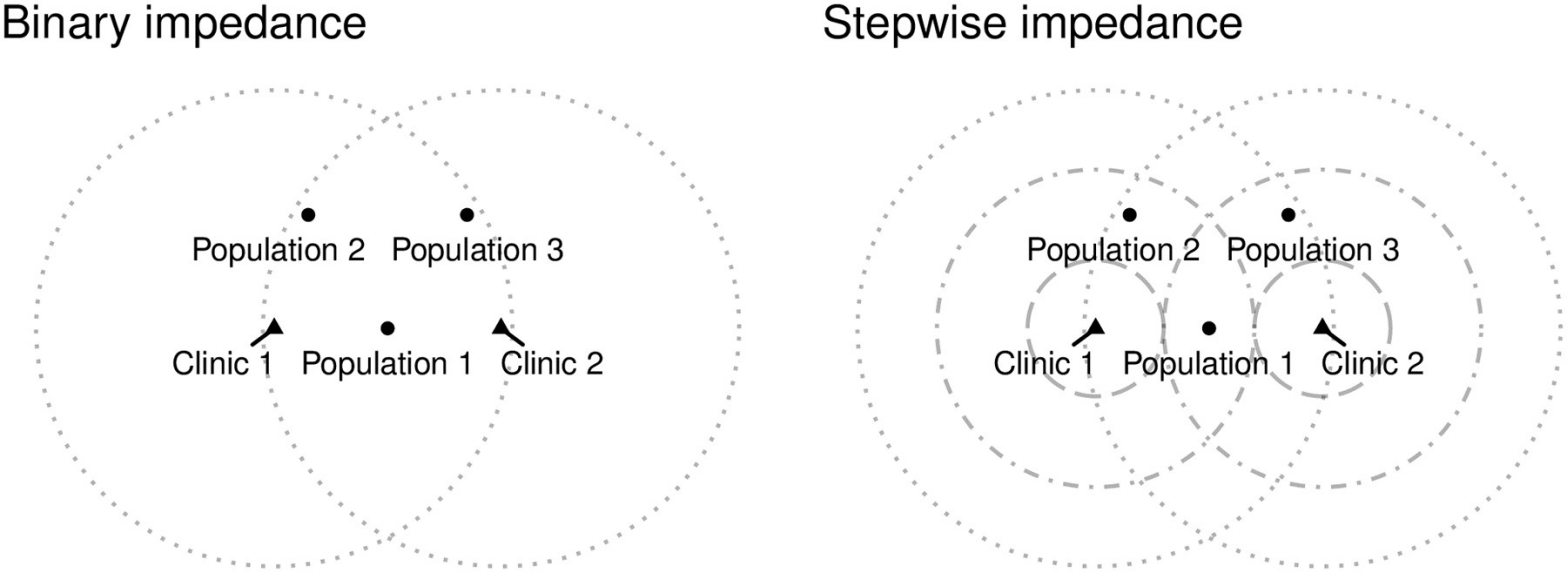
Three population centers and two clinics (triangles are clinics, dotted lines are segments of catchment areas).

Clearly, the example is too simplistic (in fact just a variation of the container approach), and it is unclear what the implications would be for a system with even just a slightly more complex geography. To explore this, consider the addition of two population centers to the landscape (see Fig 3). Notice how the three population centers are in the catchment areas of the two clinics. When the binary impedance function is used, demand at each clinic is calculated as 300, and demand over all clinics is therefore 600, or twice the population of the region. When the stepwise impedance function is used, the demand by each center is:

$$\begin{array}{c} \begin{array}{c} D_{1j}=0.8\times 100+0.8\times 100=160\\ D_{2j}=0.8\times 100+0.4\times 100=120\\ D_{3j}=0.4\times 100+0.8\times 100=120 \end{array} \end{array}$$

and the total load on the system is therefore 400, still well in excess of the total population of the region.

**Fig 4:**
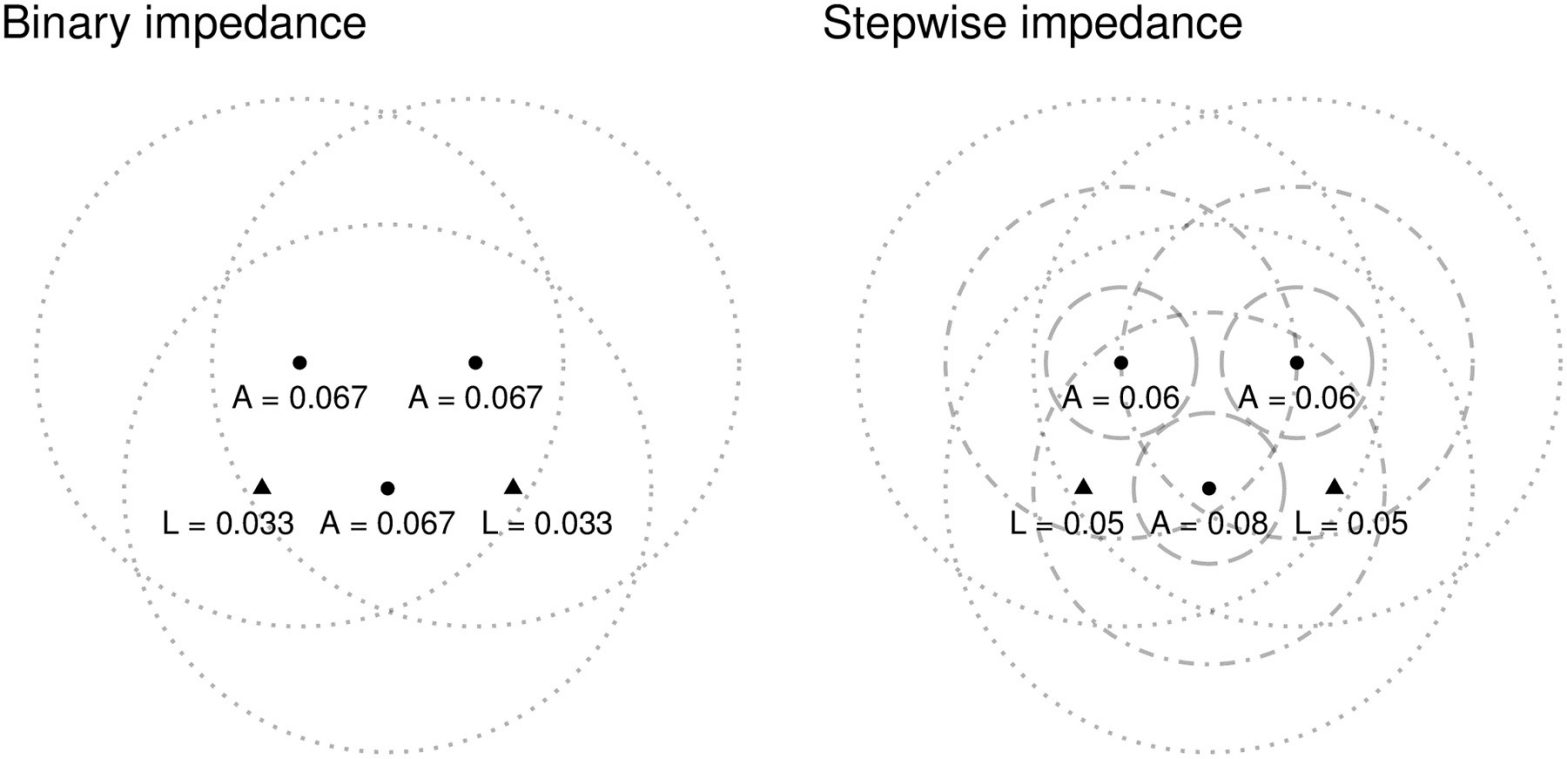
Levels of service of two clinics and accessibility of three population centers (triangles are clinics, dotted lines are segments of catchment areas).

When demand is used to calculate the level of service, and then accessibility in the second step of the algorithm, the following occurs (see Fig 4). When the binary impedance function is used (left panel), the level of service at each clinic is:

$$\begin{array}{c} \begin{array}{c} L_{1}=\frac{10}{300}=0.033\\ L_{2}=\frac{10}{300}=0.033 \end{array} \end{array}$$

The level of service at the clinics is only half of the regional PPR, since each clinic is assumed to serve the *entire* population of the region. Unfortunately, since demand has been inflated for each clinic, these levels of service cannot be meaningfully interpreted as local PPRs. The sum over the clinics, on the other hand, is 20/300—which is consistent with the regional PPR. Interestingly, as seen in the figure, the accessibility of each population center matches the regional PPR—but the sum of accessibility over all population centers exceeds the sum of the level of service over all the clinics as a consequence of allocating the same level of service to several population centers.

Continuing with the stepwise impedance function, we can see (Fig 4, right panel) that the levels of service are calculated as:

$$\begin{array}{c} \begin{array}{c} L_{1}=\frac{10}{0.8\times 100+0.8\times 100+0.4\times 100}=\frac{10}{200}=0.05\\ L_{2}=\frac{10}{0.8\times 100+0.4\times 100+0.8\times 100}=\frac{10}{200}=0.05 \end{array} \end{array}$$

Notice how the level of service is higher in this case: this is a consequence of assuming (as the stepwise impedance function does) that some of the population does *not* demand service. Demand, however, is still inflated, and interpretation of the levels of service as local PPRs is still inappropriate. Accessibility is higher for population center 1 but lower for the two peripheral centers. Furthermore, the sum of accessibility over all population centers exceeds the sum of the level of service of all clinics in the region.

At issue is the interpretability of the levels of service, which as the example illustrates do not accurately represent PPRs, and how accessibility, which is a weighted sum of levels of service, cannot be interpreted as the PPR for a population center either.

Two methods reviewed above, namely the Three-Stage Floating Catchment Area method and the Modified Two-Stage Floating Catchment Area method aim to address the overestimation of demand and/or levels of service when calculating accessibility. As discussed previously, they do this by compounding the effect of the impedance function. In the case of 3SFCA, demand is deflated by assuming that demand declines more rapidly with distance. Then, when calculating accessibility, the levels of service are allocated more locally, again, as a consequence of steeper distance-decay. In the case of M2SFCA, demand is not deflated, however, the levels of service are allocated more locally as a consequence of steeper distance-decay. In other words, these methods correct for inflation by assuming that *fewer* people demand helath care services, and that the levels of service are allocated to fewer people too.

**Fig 5:**
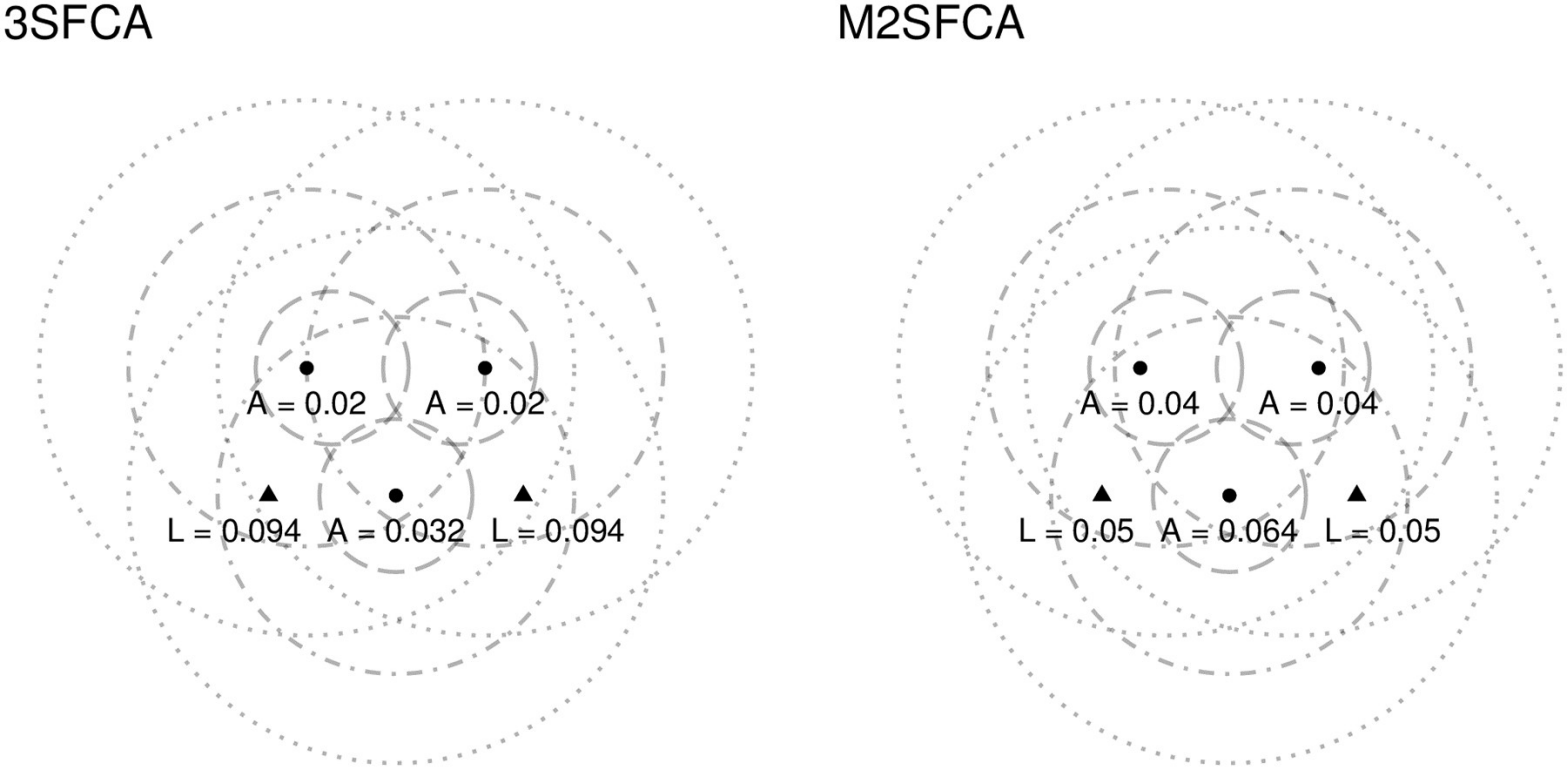
Levels of service and accessibility according to 3SFCA and M2SFCA methods (triangles are clinics, dotted lines are segments of catchment areas).

For comparison, the levels of service and accessibility for the example according to these two methods are shown in Fig 5. Notice how the levels of service in the 3FSCA are considerably higher as a consequence of excluding potential users with a steeper rate of decay. On the other hand, the levels of accessibility are also lower, as a consequence of allocating service more locally. The levels of service in the M2SFCA are identical to the E2SFCA, however, accessibility is lower, again as a result of allocating service more locally.

## A simulated example

The examples in the preceding section illustrate the way demand and level of service can be overestimaged (and in some cases underestimated) in FCA algorithms. However, they are too simplistic to indicate what would happen in a realistic situation. In particular, it is possible that the consequences depend on the geography of the problem as the examples in Delamater [18] suggest. Based on the way demand and level of service are allocated, we conjecture that the effects are likely more pronounced in areas with higher density of population and service, since inflation is a consequence of overlapping catchment areas. Furthermore, we conjecture that demand inflation will be reduced when stepwise/continuous distance-decay functions are used, since their effect is to reduce the overlap by reducing the contribution of population at different distances, and to allocate levels of service more locally as well. We explore these issues further by means of a simple but realistic simulated example.

**Fig 6:**
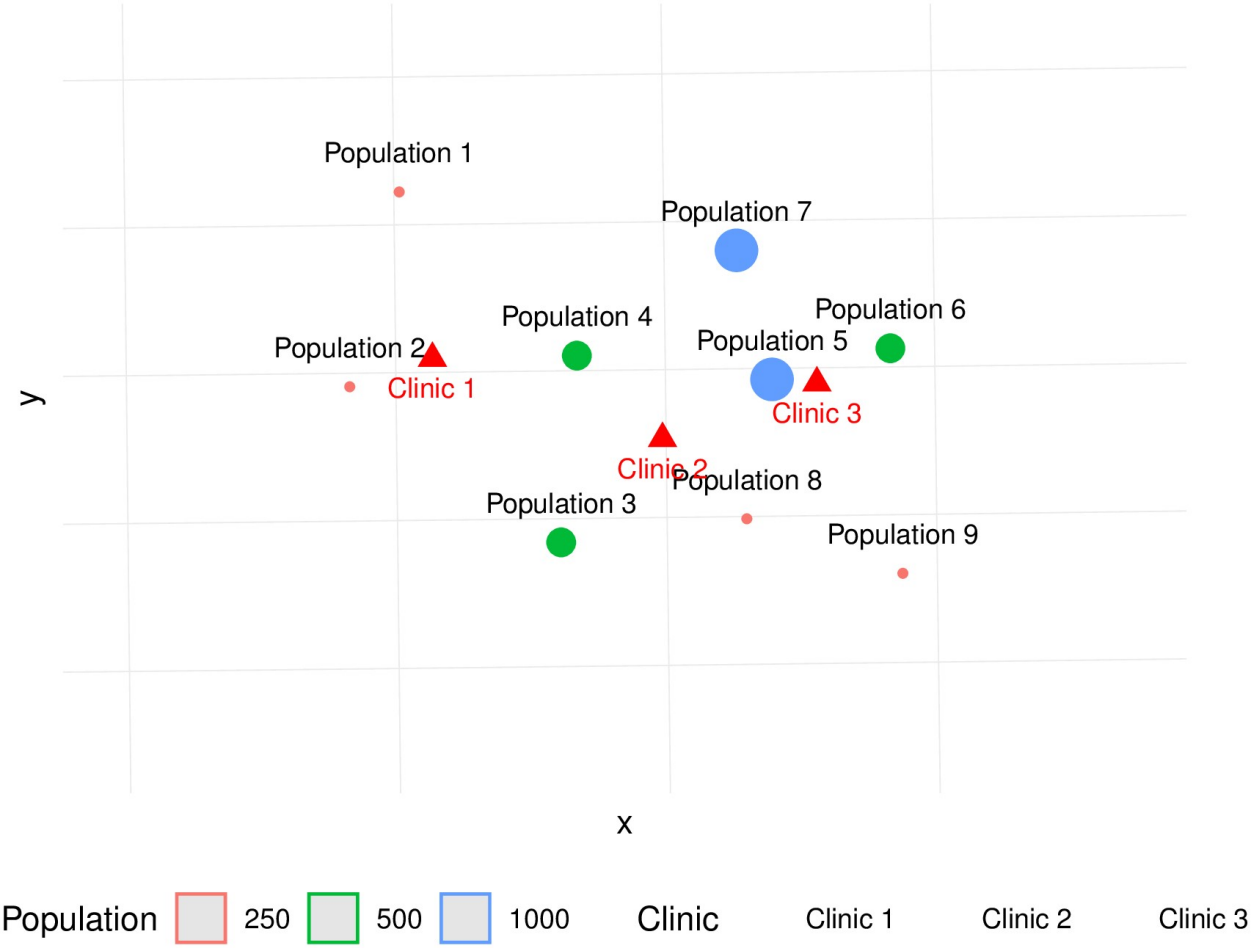
Setup for the simulation exercise.

The setup for the simulated example is shown in Fig 6. There are three clinics and nine population centers. Assume that the supply at the three clinics is one physician at clinic 1, three physicians at clinic 2, and two physicians at clinic 3. Further, assume that the population at 1, 2, 8, and 9 is 250; population at 3, 4, and 6 is 250; and population at 5 and 7 is 1000. The total population in the region therefore is 4, 500. Under this setup, the level of service across the whole system is 1.33 physicians per thousand people, which we will refer to as the Regional PPR.

**Fig 7:**
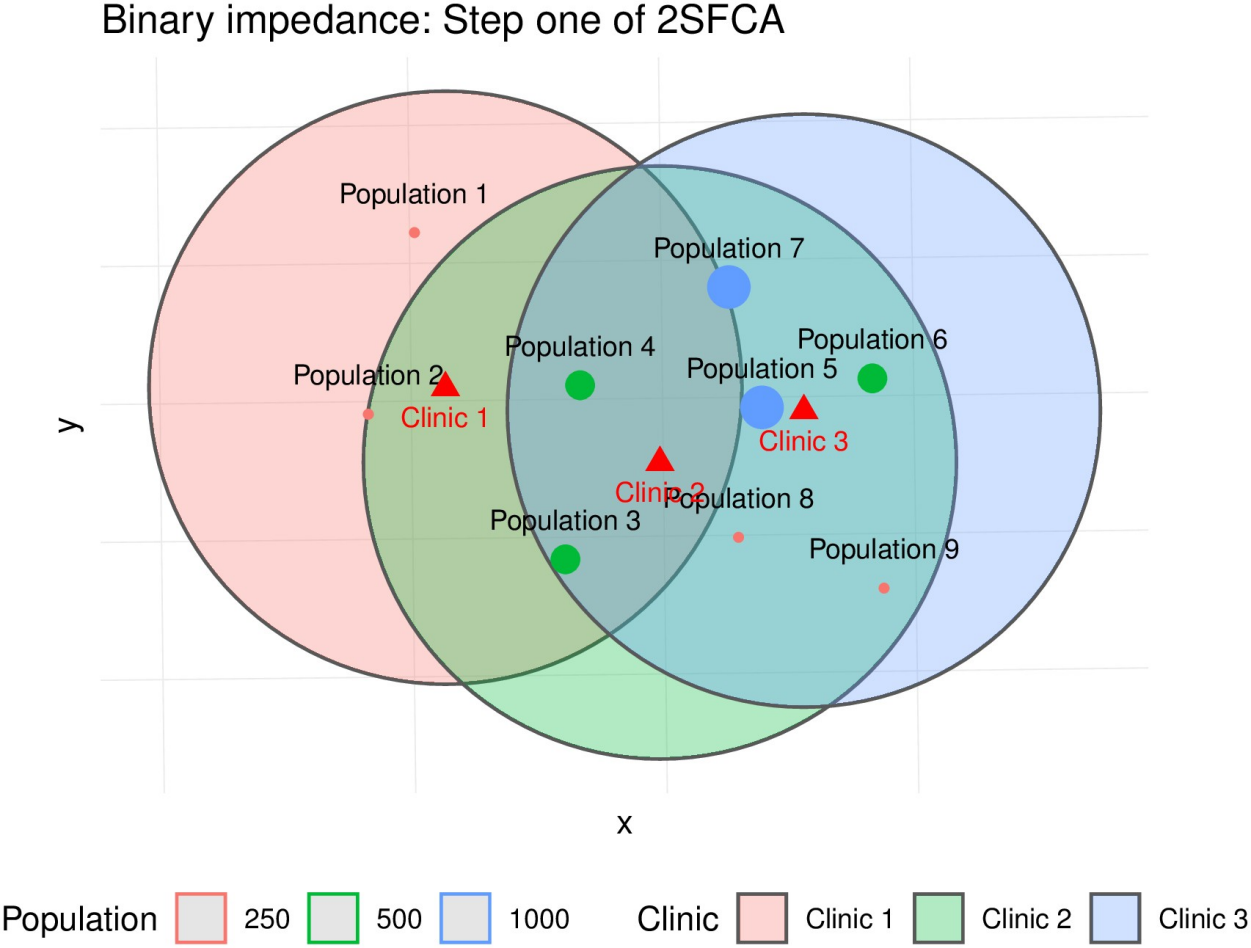
Catchment areas in step 1, according to binary and stepwise impedance functions (the weights of the stepwise function are 0.945, 0.600, and 0.424).

**Fig 8:**
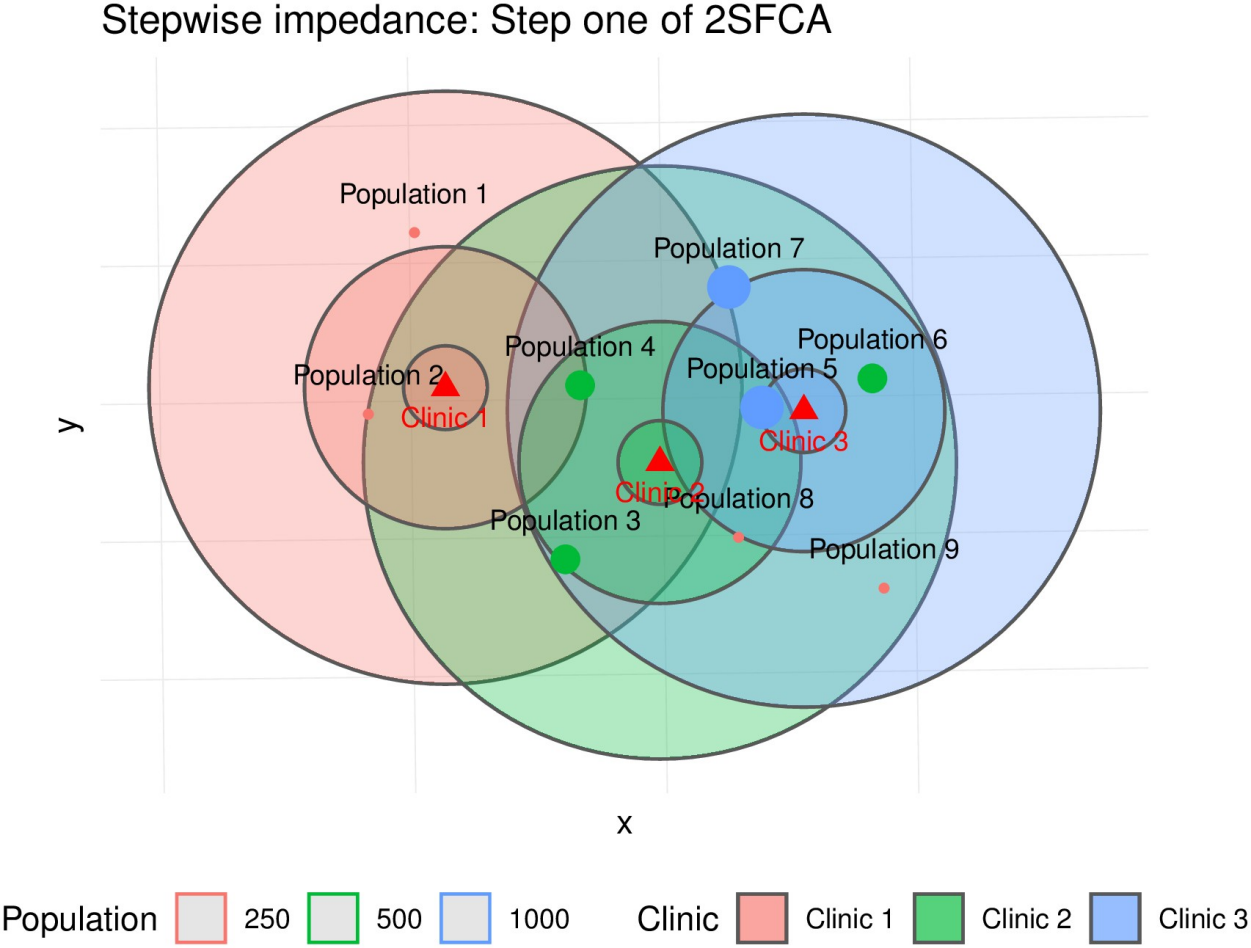
Catchment areas in step 1, according to binary and stepwise impedance functions (the weights of the stepwise function are 0.945, 0.600, and 0.424).

For this experiment, we consider binary and stepwise impedance functions. The former is simply the traditional 2SFCA method, whereas the latter is the Enhanced 2SFCA approach. The catchment areas for the first step of the algorithm (demand allocation) are shown in Fig 7 (binary impedance) and Fig 8 (stepwise impedance). Notice that some population centers are inside the catchment areas of more than one clinic. For instance, Population Center 5 is in the catchment areas of Clinics 2 and 3, whereas Population Center 4 is in the catchment areas of all three clinics.

To see how the overlap of catchment areas impacts the calculations in the first step of the algorithm, we define impedance matrices using the same criteria as for the buffers seen in Figs 7 and 8. These matrices are shown in Table 1.

**Table 1:** Impedance matrices and step 1 of the FCA algorithm.

	Binary Impedance			Stepwise Impedance		
Population Center	Clinic 1	Clinic 2	Clinic 3	Clinic 1	Clinic 2	Clinic 3
Population 1	1	0	0	0.242	0.000	0.000
Population 2	1	1	0	0.600	0.242	0.000
Population 3	1	1	1	0.242	0.600	0.242
Population 4	1	1	1	0.600	0.600	0.242
Population 5	0	1	1	0.000	0.600	0.945
Population 6	0	1	1	0.000	0.242	0.600
Population 7	0	1	1	0.000	0.242	0.242
Population 8	0	1	1	0.000	0.600	0.242
Population 9	0	1	1	0.000	0.242	0.242

The demand for each clinic is calculated as the population of the centers multiplied by the values of the corresponding impedance weight with respect to that clinic, and then aggregated for all population centers. The level of service is the supply divided by the demand, multiplied by 1, 000. The last row of the table shows the total population as well as the total demand at each clinic.

First we discuss the results according to the binary impedance function. As seen in Table 2, the population of Center 3 (which is in the catchment area of three clinics) is assumed to contribute 1, 500 patients to the demand across the system, whereas Center 1 (which is in the catchment area of only one clinic) contributes exactly its population of 250. Since the population of several centers is counted multiple times, the apparent demand exceeds the actual population. In effect, when we calculate the total demand (the sum of the demand across clinics), we find that this is 9,750 according to the binary impedance function, which far exceeds the actual population.

**Table 2:** Disaggregated demand allocations by population center and clinic, and level of service by clinic.

		Binary Impedance			Stepwise Impedance			3SFCA			M2SFCA		
Population Center	Population	Clinic 1	Clinic 2	Clinic 3	Clinic 1	Clinic 2	Clinic 3	Clinic 1	Clinic 2	Clinic 3	Clinic 1	Clinic 2	Clinic 3
Population 1	250	250	0	0	60.5	0	0	60.5	0	0	60.5	0	0
Population 2	250	250	250	0	150	60.5	0	106.89	17.388	0	150	60.5	0
Population 3	500	500	500	500	121	300	121	27.013	166.05	27.013	121	300	121
Population 4	500	500	500	500	300	300	121	124.83	124.83	20.307	300	300	121
Population 5	1000	0	1000	1000	0	600	945	0	233.01	578.01	0	600	945
Population 6	500	0	500	500	0	121	300	0	34.777	213.78	0	121	300
Population 7	1000	0	1000	1000	0	242	242	0	121	121	0	242	242
Population 8	250	0	250	250	0	150	60.5	0	106.89	17.388	0	150	60.5
Population 9	250	0	250	250	0	60.5	60.5	0	30.25	30.25	0	60.5	60.5
Total Population/Demand	4500	1500	4250	4000	631.5	1834	1850	319.228	834.191	1007.744	631.5	1834	1850
Supply	NA	1	3	2	1	3	2	1	3	2	1	3	2
Level of Service (per 1,000)	NA	0.667	0.706	0.5	1.584	1.636	1.081	3.133	3.596	1.985	1.584	1.636	1.081

Note:

Darker cell colors in a row indicate a greater allocation of demand.

Turning now to the stepwise function, we see that Center 3 contributes 500 × 0.242 + 500 × 0.600 + 500 × 0.242 = 542 to the demand across the system, but Center 1 contributes only 250 × 0.242 = 60.5. The total demand now is 4,316, which is *less* than the total population.

This example illustrates a vexing effect in how FCA methods operate: when multiple service points are within the threshold travel cost of a population center, it is assumed that some (and possibly all) of the same persons crowd more than one service point, resulting in inflated demand and deflated levels of service. On the other hand, when stepwise or continuous functions (e.g., E2SFCA) are used to weigh down the population of distant population centers, the apparent effect is that some segments of the population do *not* demand service, even when clinics are within their threshold travel cost. This effect is even more marked in the case of 3SFCA, which produces considerably higher levels of service, as a consequence of stacking the effects of two impedance functions. In effect, demand is deflated and the level of service is inflated. While the assumption that some members of the population drop out from the total demand pool may be acceptable for discretionary services, it is suspect when it comes to essential services such as many health care services, and particularly primary health care.

Recall as well that the Regional Average PPR in this example is 1.33 physicians per thousand. If the total implied demand according to the binary impedance function is 9,750 the corresponding PPR is 0.615 physicians per thousand, or about half of the regional ratio. The corresponding PPR for the stepwise impedance function (implied demand = 4315.5) is 1.39 physicians per thousand, much closer to the Regional Average PPR. However, this PPR is misleading in that it assumes that some segments of the population are served multiple times, and some are not served at all.

Clearly, the first step of the algorithm can lead to inflation or deflation of the levels of demand. But do these matter? Or do they somehow average out when the levels of service are aggregated in the second step of the algorithm? Again, the situation is not clear-cut when multiple population centers and/or service clinics interact through overlapping catchment areas.

To illustrate this, we proceed to estimate the accessibility for the example using the binary and the stepwise impedance matrices. The results appear in Table 3.

**Table 3:** Disaggregated level of service allocations by clinic and population center, and accessibility by population center.

Clinic	Supply	Demand	Level of Service	Center 1	Center 2	Center 3	Center 4	Center 5	Center 6	Center 7	Center 8	Center 9
**Binary Impedance**												
1	1	1500.000	0.667	0.667	0.667	0.667	0.667	0	0	0	0	0
2	3	4250.000	0.706	0	0.706	0.706	0.706	0.706	0.706	0.706	0.706	0.706
3	2	4000.000	0.500	0	0	0.5	0.5	0.5	0.5	0.5	0.5	0.5
Accessibility	NA	NA	NA	0.667	1.37	1.87	1.87	1.21	1.21	1.21	1.21	1.21
**Stepwise Impedance**												
1	1	631.500	1.584	0.383	0.95	0.383	0.95	0	0	0	0	0
2	3	1834.000	1.636	0	0.396	0.981	0.981	0.981	0.396	0.396	0.981	0.396
3	2	1850.000	1.081	0	0	0.262	0.262	1.02	0.649	0.262	0.262	0.262
Accessibility	NA	NA	NA	0.383	1.35	1.63	2.19	2	1.04	0.657	1.24	0.657
**3SFCA**												
1	1	319.228	3.133	0.758	1.88	0.758	1.88	0	0	0	0	0
2	3	834.191	3.596	0	0.87	2.16	2.16	2.16	0.87	0.87	2.16	0.87
3	2	1007.744	1.985	0	0	0.48	0.48	1.88	1.19	0.48	0.48	0.48
Accessibility	NA	NA	NA	0.0551	0.367	0.253	0.536	0.525	0.17	0.0514	0.198	0.0514
**M2SFCA**												
1	1	631.500	1.584	0.383	0.95	0.383	0.95	0	0	0	0	0
2	3	1834.000	1.636	0	0.396	0.981	0.981	0.981	0.396	0.396	0.981	0.396
3	2	1850.000	1.081	0	0	0.262	0.262	1.02	0.649	0.262	0.262	0.262
Accessibility	NA	NA	NA	0.0927	0.666	0.745	1.22	1.55	0.485	0.159	0.652	0.159

Note:

Darker cell colors in a column indicate a greater allocation of level of service or accessibility.

Accessibility in the table is calculated as the level of service of the clinics multiplied by the the values of the impedance function with respect to a population center, and then aggregated for all clinics. As seen in the table, the levels of accessibility vary considerably depending on the method. As anticipated, use of non-binary impedance functions reduces the inflation effect, and can even lead to deflation. Consider for instance the case of the binary impedance matrix: the total level of service in the system is the sum of the level of service at the three clinics, or 1.87. The level of service *allocated* to population centers, on the other hand, is the sum of the accessibility in the system, or 11.8. When using the stepwise impedance function, the total level of service in the system is 4.3, and the level of service allocated to population centers is 11.2. Compare this to the case of 3SFCA, where the total level of service in the system is 8.71, but the level of service allocated to population centers is only 2.21; or the case of M2SFCA, which estimates the total level of service in the system as 4.3 (same as E2SFCA) but allocates 5.74 to population centers.

**Fig 9:**
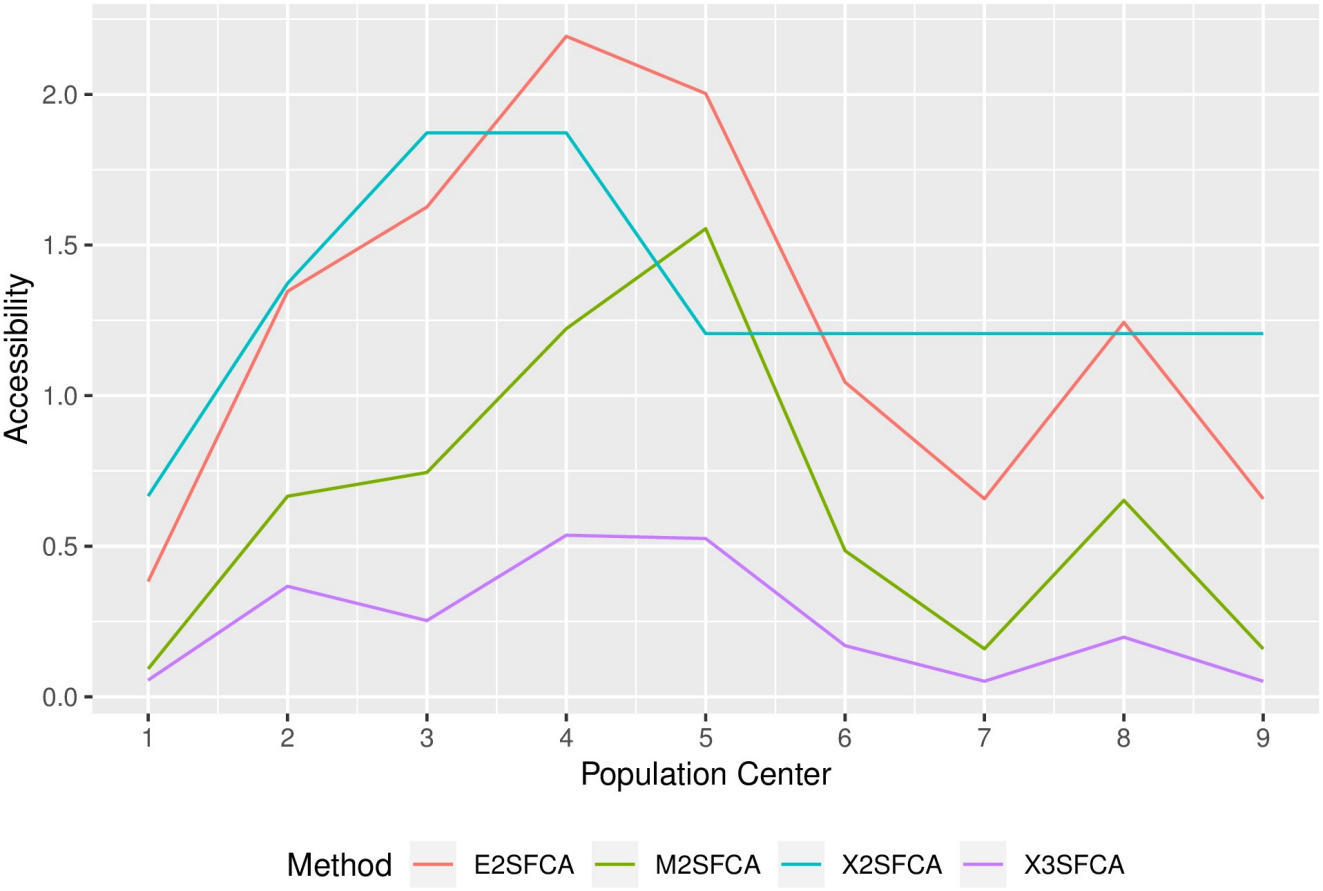
Accessibility by population center by method.

Clearly, all the methods give qualitatively similar results, with peripheral centers displaying lower accessibility and more central places higher. But there are important differences in how demand and level of service are allocated throughout the system to calculate accessibilty. Fig 9 shows how the different methods penalize peripheral centers at different rates. And, since the demand is not consistent with the population and the accessibility is not consistent with the level of service of the clinics, it is difficult to interpret the results in terms PPRs. For instance, when we inspect the results for the binary impedance matrix (2SFCA), we can see in the table that the accessibility of Population Center 1 is simply the level of service of Clinic 1. But, as we saw before, this level of service was deflated by double counting the population of Centers 2, 3, and 4, which contribute to the calculation of demand at multiple clinics. Things become more complex as the number of overlapping catchment areas grows. For example, Population Center 2 contributed to the congestion effect of two clinics. However, demand at one of those clinics was calculated using the population of eight out of nine population centers. What this suggests is that, at the very least, some population centers (likely those in the periphery of regions) will have artificially low accessibility levels as a consequence of demand inflation.

## A method for proportional allocation of demand and supply

As the examples in the preceding subsection illustrate, FCA methods can induce quite substantial inflation (or deflation) of demand and level of service. This, in turn, can affect the estimates of accessibility in potentially complex ways. The results, furthermore lack a clear interpretation. In this section, we propose a simple and intuitive adjustment to avoid the inflation artifacts inherent in current implementations of FCA methods.

Refer again to Fig 1. Demand inflation occurs because of the overlap in catchment areas—with the underlying assumption that a service location services the population within its catchment area. More realistically, only a fraction of that population will demand service at the location if other service points are within reach (i.e., inside its “floated” catchment area).

For instance, assuming (as the binary impedance function does), that individuals at Population Center 1 are indifferent between Clinics 1 and 2, then it is reasonable to think that the population will sort itself proportionally to these two clinics—in this example, this means that half of the population will attend one of two different clinics (importantly, this assumes that the services on offer are undifferentiated; one would not generally consider cancer screening and hair removal clinics competitors). This suggests the following adjustment to the way the level of demand is calculated. Given an impedance function, a set of adjusted weights, say $W_{ij}^{i*}$, are precalculated by dividing the original impedance weights by the sum of the weights for population center *i* over all service points *j*:

$$W_{ij}^{i}=\frac{W_{ij}}{\sum_{j}W_{ij}}$$

Please notice that these weights are identical to the selection weights of the 3SFCA method. A key property of the adjusted weights is the following:

$$\sum_{j}W_{ij}^{i}=1$$

This adjustment procedure has the effect that, when the level of demand of *i* is summed over all service points *j*, the aggregated level of demand due to *i* is identical to its population:

$$\sum_{j}P_{i}W_{ij}^{i}=P_{i}$$

As a result of standardizing the impedance weights, population is allocated *proportionally* to clinics.

On the supply side, inflation happens because the level of service available at location *j* is assumed to be available to every population center *i* within its catchment area. To adjust this, another set of weights, say $W_{ij}^{j*}$, is pre-calculated by dividing the original impedance weights *W*_*ij*_ by the sum of the weights for service point *j* over all population centers *i*:

$$W_{ij}^{j}=\frac{W_{ij}}{\sum_{i}W_{ij}}$$

Again, the resulting weights have the property that:

$$\sum_{i}W_{ij}^{j}=1$$

As before, the result of this procedure is that, when the level of service of *j* is aggregated by population centers, the total level of service for that service point is preserved:

$$\sum_{i}L_{j}W_{ij}^{j}=L_{j}$$

Note that, since the weights add up to one, they can be interpreted as a *probability* or *frequency* of contact, similar to the Huff model of [19].

In reference to Fig 3 (left panel), we can see that the original binary (unadjusted) weights for Population Centre 1 are *W*_11_ = 1, *W*_12_ = 1, the weights of population center 2 are *W*_21_ = 1, *W*_22_ = 1, and the weights of population center 3 are *W*_31_ = 1, *W*_32_ = 1.

On the demand side, the adjusted weights become for Population Center 1, $W_{11}^{i}=1/2$, $W_{12}^{i}=1/2$, for Population Center 2 $W_{21}^{i}=1/2$, $W_{22}^{i}=1/2$, and for Population Center 3 $W_{31}^{i}=1/2$, $W_{32}^{i}=1/2$. Using the adjusted weights, it can be seen that the level of demand due to each population center equals its respective population:

$$\begin{array}{c} \begin{array}{c} \sum_{j}D_{1}j=1/2P_{1}+1/2P_{1}=P_{1}\\ \sum_{j}D_{2}j=1/2P_{2}+1/2P_{2}=P_{2}\\ \sum_{j}D_{3}j=1/2P_{3}+1/2P_{3}=P_{3} \end{array} \end{array}$$

Coming next to the supply side, the adjusted weights for Clinic 1 are $W_{11}^{j*}=1/3$, $W_{21}^{i*}=1/3$, and $W_{23}^{i*}=1/3$; for Clinic 2 the adjusted weights are $W_{12}^{j*}=1/3$, $W_{22}^{i*}=1/3$, and $W_{32}^{i*}=1/3$. It can be seen that the level of service is preserved across clinics, and therefore across the system:

$$\begin{array}{c} \begin{array}{c} \sum_{i}L_{i1}=L_{1}/3+L_{1}/3+L_{1}/3=L_{1}\\ \sum_{i}L_{i2}=L_{2}/3+L_{2}/3+L_{2}/3=L_{2} \end{array} \end{array}$$

The method to adjust the weights used above is identical to a procedure that will be familiar to readers acquainted with the literature in the fields of spatial statistics and econometrics. The same adjustment is widely used there under the names of row- and column-standardization of a weights matrix [24, 25].

The proposed adjustment can be easily implemented. We will present next the implementation using a compact matrix notation. Begin by defining the following impedance matrix:

$$\begin{array}{c} W=(\begin{array}{ccc} W_{11} & \cdots & W_{1J}\\ \vdots & \ddots & \vdots \\ W_{N1} & \cdots & W_{NJ} \end{array}) \end{array}$$

where *W*_*ij*_ is an impedance function evaluated at *d*_*ij*_. Subindex *i* is for population centers (*i* = 1, …, *N*) and subindex *j* is for service points (*j* = 1, …, *J*). Note that the matrix does not need to be square. A row-standardized set of weights is obtained as follows:

$$\begin{array}{c} W^{i}=(\begin{array}{ccc} \frac{W_{11}}{\sum_{j}W_{1j}} & \cdots & \frac{W_{1J}}{\sum_{j}W_{1j}}\\ \vdots & \ddots & \vdots \\ \frac{W_{N1}}{\sum_{j}W_{Nj}} & \cdots & \frac{W_{NJ}}{\sum_{j}W_{Nj}} \end{array}) \end{array}$$

Next, a column-standardized set of weights is calculated as:

$$\begin{array}{c} W^{j}=(\begin{array}{ccc} \frac{W_{11}}{\sum_{i}W_{i1}} & \cdots & \frac{W_{1J}}{\sum_{i}W_{iJ}}\\ \vdots & \ddots & \vdots \\ \frac{W_{N1}}{\sum_{i}W_{i1}} & \cdots & \frac{W_{NJ}}{\sum_{i}W_{iJ}} \end{array}) \end{array}$$

In the first example above (see Fig 1), the binary impedance matrix is:

$$\begin{array}{c} W_{binary}=(\begin{array}{cc} 1 & 1\\ 1 & 1\\ 1 & 1 \end{array}) \end{array}$$

The row-standardized weights that correspond to this matrix are:

$$\begin{array}{c} W_{binary}^{i}=(\begin{array}{cc} 1/2 & 1/2\\ 1/2 & 1/2\\ 1/2 & 1/2 \end{array}) \end{array}$$

and the column-standardized weights are:

$$\begin{array}{c} W_{binary}^{j}=(\begin{array}{cc} 1/3 & 1/3\\ 1/3 & 1/3\\ 1/3 & 1/3 \end{array}) \end{array}$$

The stepwise impedance weights in the example are:

$$\begin{array}{c} W_{stepwise}=(\begin{array}{cc} 0.8 & 0.8\\ 0.8 & 0.4\\ 0.4 & 0.8 \end{array}) \end{array}$$

The row-standardized weights in turn are:

$$\begin{array}{c} W_{stepwise}^{i}=(\begin{array}{cc} 1/2 & 1/2\\ 2/3 & 1/3\\ 1/3 & 2/3 \end{array}) \end{array}$$

whereas the column-standardized weights are:

$$\begin{array}{c} W_{stepwise}^{j}=(\begin{array}{cc} 4/10 & 4/10\\ 4/10 & 2/10\\ 2/10 & 4/10 \end{array}) \end{array}$$

Once that the impedance weights have been adjusted, a vector of adjusted level of demand D* can be obtained by multiplying the *transposed* impedance matrix by a vector of population values as follows:

$$D^{*}={\left[W^{i}\right]}^{T}P$$

where the ^*T*^ operator is for “transpose”, and **P** is:

$$\begin{array}{c} P=(\begin{array}{c} P_{1}\\ \vdots \\ P_{N} \end{array}) \end{array}$$

The level of demand for the service points in the binary impedance function example is (in vector form):

$$\begin{array}{c} D_{binary}^{*}=(\begin{array}{ccc} 1/2 & 1/2 & 1/2\\ 1/2 & 1/2 & 1/2 \end{array})(\begin{array}{c} 100\\ 100\\ 100 \end{array})=(\begin{array}{c} 300/2\\ 300/2 \end{array})=(\begin{array}{c} 150\\ 150 \end{array}) \end{array}$$

Notice how each clinic is expected to service only 150, and the level of demand over the system is identical to the total population.

The level of demand for the service points in the stepwise impedance function example is (in vector form):

$$\begin{array}{c} D_{sw}^{*}=(\begin{array}{ccc} 1/2 & 2/3 & 1/3\\ 1/2 & 1/3 & 2/3 \end{array})(\begin{array}{c} 100\\ 100\\ 100 \end{array})=(\begin{array}{c} 50+200/3+100/3\\ 50+100/3+200/3 \end{array})=(\begin{array}{c} 150\\ 150 \end{array}) \end{array}$$

As can be seen, the aggregated level of demand, after the adjustment, equals (as desired) the actual population of the region. In the case of the stepwise function, total demand has been adjusted to the population of the region without the restrictive assumption that some people are excluded from the system. This is achieved by assuming an assortative process that leads to proportional allocation of the demand.

The levels of demand can then be used to calculate the level of service at the individual clinic locations by performing Hadamard division (⊘) of the vector of supply by the vector of adjusted demand. This is the first step of the 2SFCA (aggregating demand over catchment areas for service points):

$$L^{*}=S⊘D^{*}$$

Since Hadamard division is an element-by-element operation, the adjusted levels of service in the first example (using the binary impedance function) are:

$$\begin{array}{c} L_{b}^{*}=(\begin{array}{c} 10\\ 10 \end{array})⊘(\begin{array}{c} 150\\ 150 \end{array})=(\begin{array}{c} 10/150\\ 10/150 \end{array})=(\begin{array}{c} 0.067\\ 0.067 \end{array}) \end{array}$$

The levels of service in the second example, when using the stepwise impedance function, are also:

$$\begin{array}{c} L_{sw}^{*}=(\begin{array}{c} 10\\ 10 \end{array})⊘(\begin{array}{c} 150\\ 150 \end{array})=(\begin{array}{c} 10/150\\ 10/150 \end{array})=(\begin{array}{c} 0.067\\ 0.067 \end{array}) \end{array}$$

Unlike the 2SFCA, E2SFCA, and 3SFCA methods that produce levels of service that resemble PPRs but with values that are inconsistent with total demand given the population, this operation returns values that are genuinely local PPRs that are consistent with the population of the region. As we saw above, the demand equals the population. Here, the supply also equals the number of physicians in the region. Because both demand and supply are not inflated or deflated in this rectified method, these values are easily interpretable relative to the Regional Average PPR of 20/300 or 0.067 physicians per person. In the case of the example, it is clear that both clinics have PPRs that are identical to the Regional Average PPR.

Accessibility, finally, is calculated as the matrix product of the column-standardized weights and the adjusted level of service:

$$A^{*}=W^{j}L^{*}$$

which, continuing with the example, gives the following for the binary impedance function:

$$\begin{array}{c} A_{b}^{*}=(\begin{array}{cc} 1/3 & 1/3\\ 1/3 & 1/3\\ 1/3 & 1/3 \end{array})(\begin{array}{c} 10/150\\ 10/150 \end{array})=(\begin{array}{c} 10/450+10/450\\ 10/450+10/450\\ 10/450+10/450 \end{array})=(\begin{array}{c} 0.044\\ 0.044\\ 0.044 \end{array}) \end{array}$$

Notice how the sum of accessibility over the region is consistent with the total level of service over all clinics (i.e., 0.133). The level of service has been allocated in its totality.

When using the stepwise impedance function, accessibility is calculated as:

$$\begin{array}{c} A_{sw}^{*}=(\begin{array}{cc} 4/10 & 4/10\\ 4/10 & 2/10\\ 2/10 & 4/10 \end{array})(\begin{array}{c} 10/150\\ 10/150 \end{array})=(\begin{array}{c} 4/150+4/150\\ 4/150+2/150\\ 2/150+4/150 \end{array})=(\begin{array}{c} 0.053\\ 0.040\\ 0.040 \end{array}) \end{array}$$

Again, the sum of accessibility is consistent with the level of service available from all clinics in the region. As with the Local PPRs, accessibility is interpreted as population-to-provider ratios for each population center in such a way that all calculations are with total demand and total level of service. In particular, accessibility can be interpreted as the share of level of service that a population center receives from all the clinics that service it.

For the sake of comparison, levels of service and accessibility are reported for the simulated example in Tables 4 and 5.

**Table 4:** Disaggregated proportional demand allocations by population center and clinic, and level of service by clinic, using adjusted weights.

		Binary Impedance—Adjusted			Stepwise Impedance—Adjusted		
Population Center	Population	Clinic 1	Clinic 2	Clinic 3	Clinic 1	Clinic 2	Clinic 3
Population 1	250	250	0	0	250	0	0
Population 2	250	125	125	0	178.15	71.853	0
Population 3	500	166.67	166.67	166.67	111.62	276.75	111.62
Population 4	500	166.67	166.67	166.67	208.04	208.04	83.911
Population 5	1000	0	500	500	0	388.35	611.65
Population 6	500	0	250	250	0	143.71	356.29
Population 7	1000	0	500	500	0	500	500
Population 8	250	0	125	125	0	178.15	71.853
Population 9	250	0	125	125	0	125	125
Total Population/Demand	4500	708.333	1958.333	1833.333	747.815	1891.852	1860.333
Supply	NA	1	3	2	1	3	2
Level of Service (per 1,000)	NA	1.412	1.532	1.091	1.337	1.586	1.075

Note:

Darker cell colors in a row indicate a greater allocation of demand.

**Table 5:** Disaggregated proportional level of service allocations by clinic and population center, and accessibility by population center, using adjusted weights.

Clinic	Supply	Demand	Level of Service	Center 1	Center 2	Center 3	Center 4	Center 5	Center 6	Center 7	Center 8	Center 9
**Binary Impedance—Adjusted**												
1	1	708.333	1.412	0.353	0.353	0.353	0.353	0	0	0	0	0
2	3	1958.333	1.532	0	0.191	0.191	0.191	0.191	0.191	0.191	0.191	0.191
3	2	1833.333	1.091	0	0	0.156	0.156	0.156	0.156	0.156	0.156	0.156
Accessibility	NA	NA	NA	0.353	0.544	0.7	0.7	0.347	0.347	0.347	0.347	0.347
**Stepwise Impedance—Adusted**												
1	1	747.815	1.337	0.192	0.476	0.192	0.476	0	0	0	0	0
2	3	1891.852	1.586	0	0.114	0.282	0.282	0.282	0.114	0.114	0.282	0.114
3	2	1860.333	1.075	0	0	0.0944	0.0944	0.369	0.234	0.0944	0.0944	0.0944
Accessibility	NA	NA	NA	0.192	0.59	0.569	0.853	0.651	0.348	0.208	0.377	0.208

Note:

Darker cell colors in a column indicate a greater allocation of level of service or accessibility.

An important point to remark is the following. The use of row- and column-standardized impedance weights assumes that the full population of every population center within the catchment of a clinic will receive service. However, the allocation, although proportional, is different when binary or stepwise impedance weights are standardized. When binary weights are employed, the underlying idea is that potential for use is identical within the catchment area irrespective of distance. When stepwise weights are used, proportionally more of the population is allocated to closer clinics. Depending on the definition of cost of travel, this allows a research to accommodate directional effects as well. For example, use of network travel time would tend to favor movement away from congested locations.

### Suboptimal systems

The research of Delamater [18] illustrates how accessibility estimates can be misleading when systems are not optimally configured. We understand this to mean that some population centers are located too far away from service points to actually benefit from them. In the modified 2SFCA method (M2SFCA), Delamater addresses this issue by increasing the friction of distance. A slight inconsistency in this approach is that some of the centers that contribute to demand fail to benefit from the service due to the increased friction to which the allocation of the level of service is subjected. Our suggestion in the case of suboptimal systems is to use an impedance function that reflects limiting conditions. For instance, in urban settings a travel time longer than 2 hours might be considered too long to be serviced by any clinic.

### System efficiency

The approach proposed in this paper allocates population and level of service proportionally and exactly. This assumes that the population sorts itself into clinics in the most efficient way. But what if some members of the population lack full information about the spatial distribution of clinics? Or have some bias towards centric locations? The vagaries of human behavior could create excess demand in some locations, and as a consequence supply surpluses in others. Situations like this can be accommodated in a relatively straightforward way using our approach.

Here, we describe the use of *slack factors*. Demand and level of service are allocated proportionally and exhaustively (i.e., 100%). But the standardization could allow for some slack, by inflating demand and/or supply in a controlled way.

Our proposal to standardize the weights was as follows, for the case of rows and columns respectively:

$$\begin{array}{c} W^{i}=(\begin{array}{ccc} \frac{W_{11}}{\sum_{j}W_{1j}} & \cdots & \frac{W_{1J}}{\sum_{j}W_{1j}}\\ \vdots & \ddots & \vdots \\ \frac{W_{N1}}{\sum_{j}W_{Nj}} & \cdots & \frac{W_{NJ}}{\sum_{j}W_{Nj}} \end{array})\;\text{and}\;W^{j}=(\begin{array}{ccc} \frac{W_{11}}{\sum_{i}W_{i1}} & \cdots & \frac{W_{1J}}{\sum_{i}W_{iJ}}\\ \vdots & \ddots & \vdots \\ \frac{W_{N1}}{\sum_{i}W_{i1}} & \cdots & \frac{W_{NJ}}{\sum_{i}W_{iJ}} \end{array}) \end{array}$$

A set of slack factors, say $k_{i}^{i}$, could be introduced in the following manner:

$$\begin{array}{c} W^{i}=(\begin{array}{ccc} \frac{k_{1}^{i}W_{11}}{\sum_{j}W_{1j}} & \cdots & \frac{k_{1}^{i}W_{1J}}{\sum_{j}W_{1j}}\\ \vdots & \ddots & \vdots \\ \frac{k_{N}^{i}W_{N1}}{\sum_{j}W_{Nj}} & \cdots & \frac{k_{N}^{i}W_{NJ}}{\sum_{j}W_{Nj}} \end{array}) \end{array}$$

A value of $k_{1}^{i}=1.10$ would inflate the demand of population center *i* = 1 by 10%, whereas a value of $k_{1}^{i}=1.20$ would inflate the demand by 20%. In a similar way, a set of slack factors $k_{i}^{j}$ could be introduced to modulate the allocation of supply:

$$\begin{array}{c} W^{j}=(\begin{array}{ccc} \frac{k_{1}^{j}W_{11}}{\sum_{i}W_{i1}} & \cdots & \frac{k_{J}^{j}W_{1N_{J}}}{\sum_{i}W_{iJ}}\\ \vdots & \ddots & \vdots \\ \frac{k_{1}^{j}W_{N1}}{\sum_{i}W_{i1}} & \cdots & \frac{k_{J}^{j}W_{NJ}}{\sum_{i}W_{iJ}} \end{array}) \end{array}$$

A value of $k_{1}^{j}=0.9$, for example, would deflate the supply of clinic *j* = 1 by 10%. The use of slack factors provides an interesting way of modulating demand and level of service allocation in a very precise and controlled way, and presents interesting opportunities as well to introduce expert opinion or other empirical approaches to callibrate slack factors.

## Empirical example

In the reminder of the paper we present an empirical example to illustrate the application of the methods presented above. Based on the preceding discussion, the adjusted 2SFCA method employed can be summarized as:

$$L_{j}=\sum_{i}\frac{S_{j}}{P_{i}W_{ij}^{i}}$$

with row-standardized impedance weights $W_{ij}^{i}$ in the first step, and:

$$A_{i}=\sum_{j}L_{j}W_{ij}^{j}$$

with colum-standardized impedance weights $W_{ij}^{j}$ in the second step. The same approach is used to re-weight the impedance function for the stepwise approach (i.e., E2SFCA).

The case study is based on accessibility to family physicians in the Hamilton Census Metropolitan Area (CMA), in Ontario, Canada. For this, we use data collected about the distribution of the population and primary health care clinics (i.e., family physicians) in the region. Time use data from Canada’s General Social Survey (GSS) was also used to inform the selection of thresholds for the impedance functions. The data collection and preprocessing protocols are described next.

### Family physicians and clinic locations

In regards to the supply of clinics, the locations of family physicians were obtained using the College of Physicians and Surgeons of Ontario (CPSO) database for the Province of Ontario. We chose this organization beacuse all physicians practicing in Ontario are required to register with the CPSO, as set out in the Ontario Regulation 865/93: Registration [26].

Our search of CPSO’s database was conducted attending to the following criteria.

1. Only physicians who are registered as family physicians were selected (this excluded specialists such as pediatric physicians).
2. The spatial extent of the search was determined using forward sortation areas (FSAs), which are the first three initial characters of a postal code. Using a GIS, the regions of interest were selected by choosing FSAs within a 10 kilometer buffer distance from the Hamilton CMA boundary. This involved 72 different FSA regions. Each FSA region code was then searched in the CPSO database in addition to the family physician specification.

**Fig 10:**
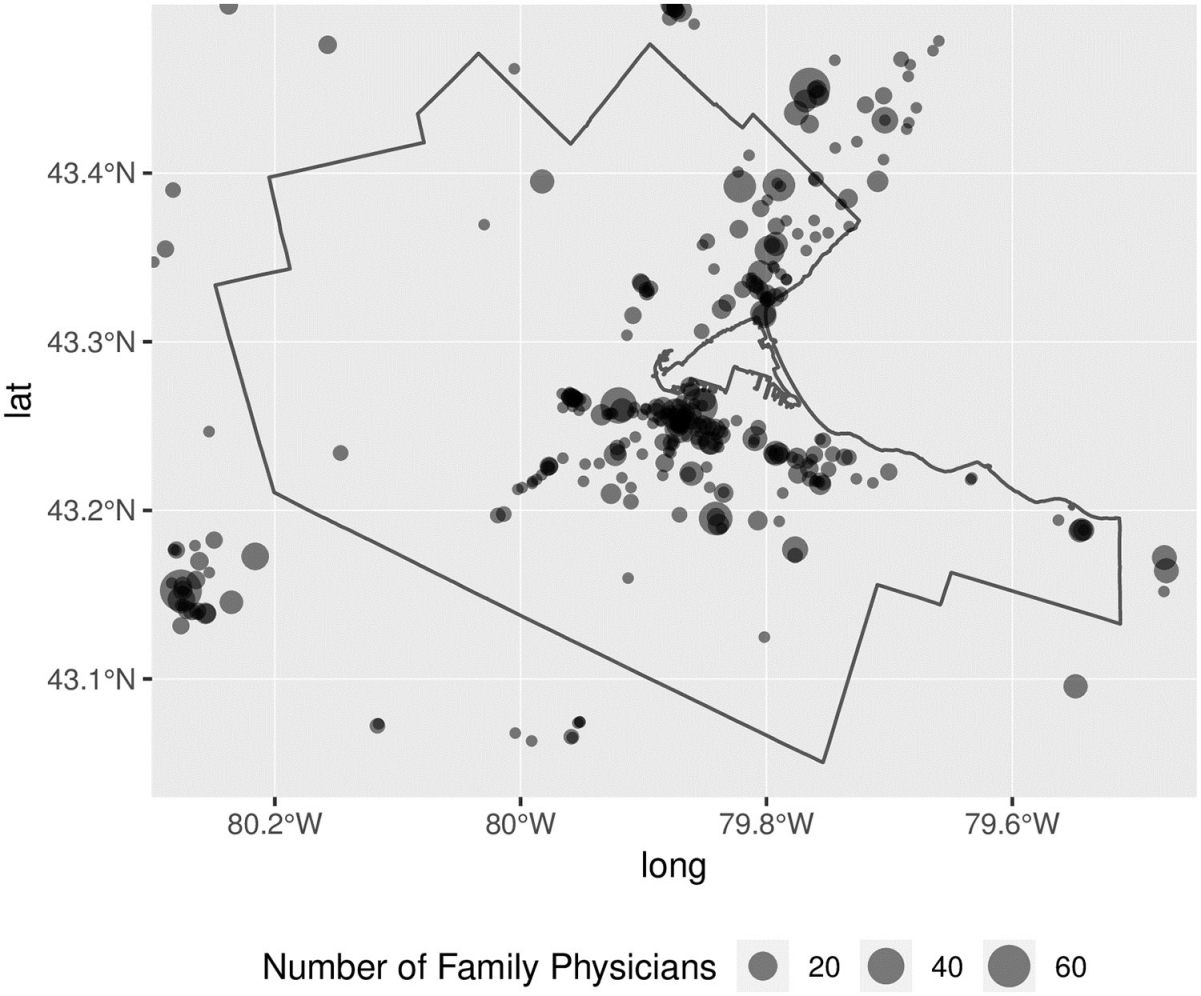
Location of clinics and family physicians in the Hamilton CMA and surrounding regions.

The parameters of the search were deliberately conservative, and the search identified a total of 2,224 family physicians practicing in the region, of which, 864 are located in the Hamilton CMA. The resulting dataset was manually verified by the third author to ensure that the information was consistent and suitable for geocoding. Prior to geocoding, locational information was organized in three columns, containing street address, city name, and province name. After family physicians were geocoded, locations were further examined. When family physicians overlapped or were within a 50 meter distance of each other we merged the records to identify 535 unique locations that we term “clinics”. Many of these clinics are not in the Hamilton CMA proper, but provide a buffer to minimize edge effects in the analysis. The distribution of clinics and family physicians is shown in Fig 10 for the Hamilton CMA.

### Population

**Fig 11:**
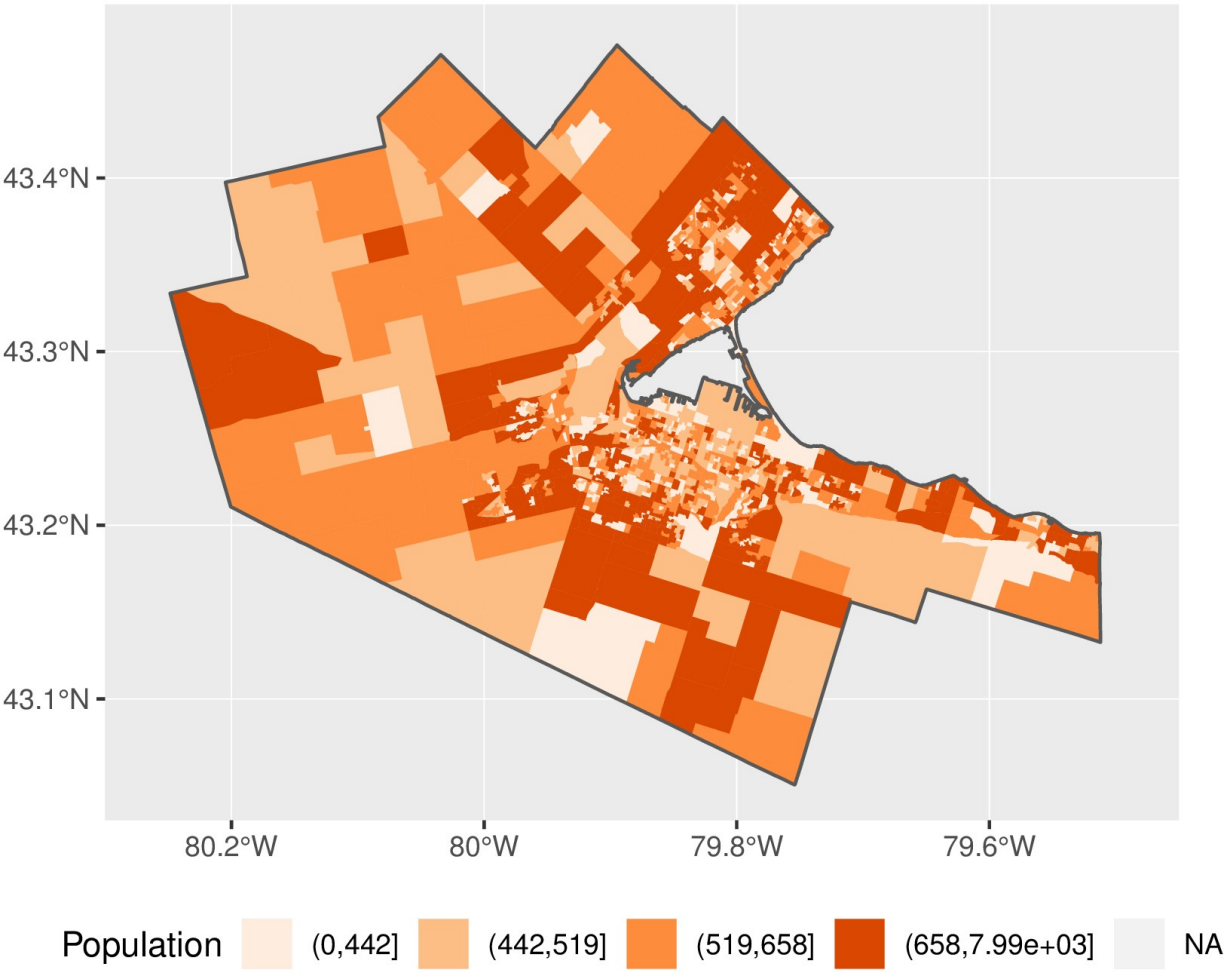
Population distribution in the Hamilton CMA.

Population information was obtained from the 2011 Canadian Census. To maximize the spatial resolution, population data were acquired at the Dissemination Area (DA) level of geography for all DAs within the selected FSAs. As a result, this includes DAs not in the Hamilton CMA proper, but that provide a buffer against edge effects. From this, the region contains a population of 2,959,090, of which 720,725 are in the Hamilton CMA. The distribution of population in the Hamilton CMA is shown in Fig 11.

### Travel time matrix

Calculation of impedance weights requires that we evaluate an impedance function at values of *d*_*i*_ *j*, that is, the cost of travel between DA *i* and clinic *j*. In this research we used travel time as our cost variable. To this end, we computed a matrix of travel times measured over the road network. To calculate the travel time between population centers and clinics we used the DA centroids and the geocoded locations of clinics. Shortest paths on the network and subsequently travel times were computed using a Geographic Information System.

### Impedance functions

For the experiments we use two different impedance functions, corresponding to the 2SFCA and E2SFCA algorithms. We do not implement the 3SFCA or the M2SFCA methods because, as noted above, they are equivalent to using steeper impedances. For the 2SFCA apprach, impedance is given by a binary function, whereas for E2SFCA it is given by a stepwise function. The impedance functions require that we define cost (i.e., travel time) thresholds to implement them. To select the thresholds, we retrieved time use data from Canada’s General Social Survey Cycle 24 (see http://odesi2.scholarsportal.info/webview/).

From the time use files, we filtered all activity episodes corresponding to respondents living in CMAs/CAs (metropolitan regions) in Ontario. Next, we filtered all episodes taking place in a car (as driver) while traveling for personal care activities for household adults (which includes traveling to see a doctor) and traveling for shopping or obtaining services (which includes traveling to go to health clinic or doctor’s office). It is worthwhile noting that travel by car accounts for over 95% of trips for the selected purposes in Ontario CMAs/CAs.

Once episodes were filtered by mode of travel and purpose of the trip, their durations (in minutes) were examined by means of quantile analysis, using episode weights to ensure the representativeness of the analysis. From the results, we learned that 50% of all trips by car for the aforementioned purposes are less than 15 minutes long, and we selected this value as the threshold *d*_0_ for the binary function. In other words, this part of the analysis assumes that any person who has to travel longer than 15 minutes to reach a clinic is outside its catchment area. We deem this value appropriate for the scale, density, and level of congestion of Hamilton CMA.

Quantile analysis of trip durations was also used to calibrate a Gaussian function with standard deviation set at 15 minutes, to match the value selected for the binary impedance above. This produced the following stepwise function, with any trips longer than 45 minutes assumed to be outside of catchment:

$$W\left(d_{ij}\right)=\{\begin{array}{cc} 0.946 & \;d_{ij}\leq 5\\ 0.801 & \;5<d_{ij}\leq 10\\ 0.607 & \;10<d_{ij}\leq 15\\ 0.411 & \;15<d_{ij}\leq 20\\ 0.135 & \;20<d_{ij}\leq 30\\ 0.011 & \;30<d_{ij}\leq 45\\ 0.00 & \;45<d_{ij} \end{array}$$

Notice how the stepwise function has weights greater than 0.5 for *d*_*ij*_ ≤ 15*min* and less than 0.5 for *d*_*ij*_ > 15*min*. This means that it will count fewer people than the binary function when *d*_*ij*_ ≤ 15*min*, but more when *d*_*ij*_ > 15*min*.

### Results

We begin our discussion of the results by noting that with a total population of the region of 2,959,090 and 2,222 family physicians, the Regional Average PPR ratio is 0.751 family physicians per 1,000 people. This value is somewhat lower than the value of 1.16 for Ontario reported by CIHI [27] and lower than the 1.20 estimated based on the population and physician data for the Hamilton CMA, which we attribute to our conservative search criteria of family physicians in the rest of the region.

The nominal levels of demand, service, and accessibility are calculated for the 2SFCA and E2SFCA using both the unadjusted and adjusted impedance matrices. Table 6 summarizes the results by each impedance matrix. As seen there, when no adjustment is made, the nominal demand explodes to several times the actual population in the region. However, when the impedance weights are standardized, demand is now only slightly less than the total population for the region, since the system is not optimal in the sense discussed by Delamater [18], and a small proportion of the population turns out to be outside of catchment. The nominal demand under binary impedance is lower due to the stricter catchment area condition (i.e., less than 15 minutes), compared to the stepwise function (i.e., less than 45 minutes). This, in turn, is somewhat lower than the total demand in the Regional Average PPR, which does not impose catchment area constraints within the region.

**Table 6:** Summary of results accessibility analysis Hamilton CMA.

Case	Supply (Doctors)	Nominal Demand	Regional Average PPR	Mean Level of Service per Clinic	Total Level of Service	Mean Accessibility per DA	Total Accessibility
Binary	2222	176,244,020	0.013	0.025	14.766	0.770	3481.709
Binary Adjusted	2222	2,861,445	0.777	1.046	607.803	0.134	607.803
Stepwise	2222	209,666,501	0.011	0.069	40.050	0.773	3499.115
Stepwise Adjusted	2222	2,957,095	0.751	0.879	510.663	0.113	510.663

It is clear that the rectified demand leads to results that are considerably more realistic than the conventional approaches. In addition to the nominal system-wide demand, this is seen as well when calculating the regional provider-to-population ratios for each case (i.e., Family Physicians per 1,000 people). As seen in the table, the mean levels of service for clinics in the region in the case of the adjusted binary and stepwise weights are in line with their corresponding Regional Average PPRs. Since the levels of service in the case of the adjusted weights can be interpreted as local PPRs, this indicates that the average clinic offers approximately the same level of service as the regional system does for the whole population. Furthermore, the mean accessibility of a DA according to the adjusted weights is identical to the mean LOS: this is because the LOS is allocated completely to DAs. The total LOS and accessibility in the region match when the adjusted weights are used. This is not the case when the unadjusted weights are used. Clearly, the use of the unadjusted weights can lead to a substantial amount of accessibility inflation, by factors as high as five or six times the estimates of the proposed proportional allocation approach.

These results demonstrate how inflation of the supply (i.e., the level of service) leads to much higher values of accessibility in the case of the conventional 2SFCA and E2SFCA methods. The procedure to rectify the population and level of service, on the other hand, leads to accessibility outputs that are consistent with the regional population and overall supply of health care services. This, in turn, makes interpretation of the output more robust and intuitive.

**Fig 12:**
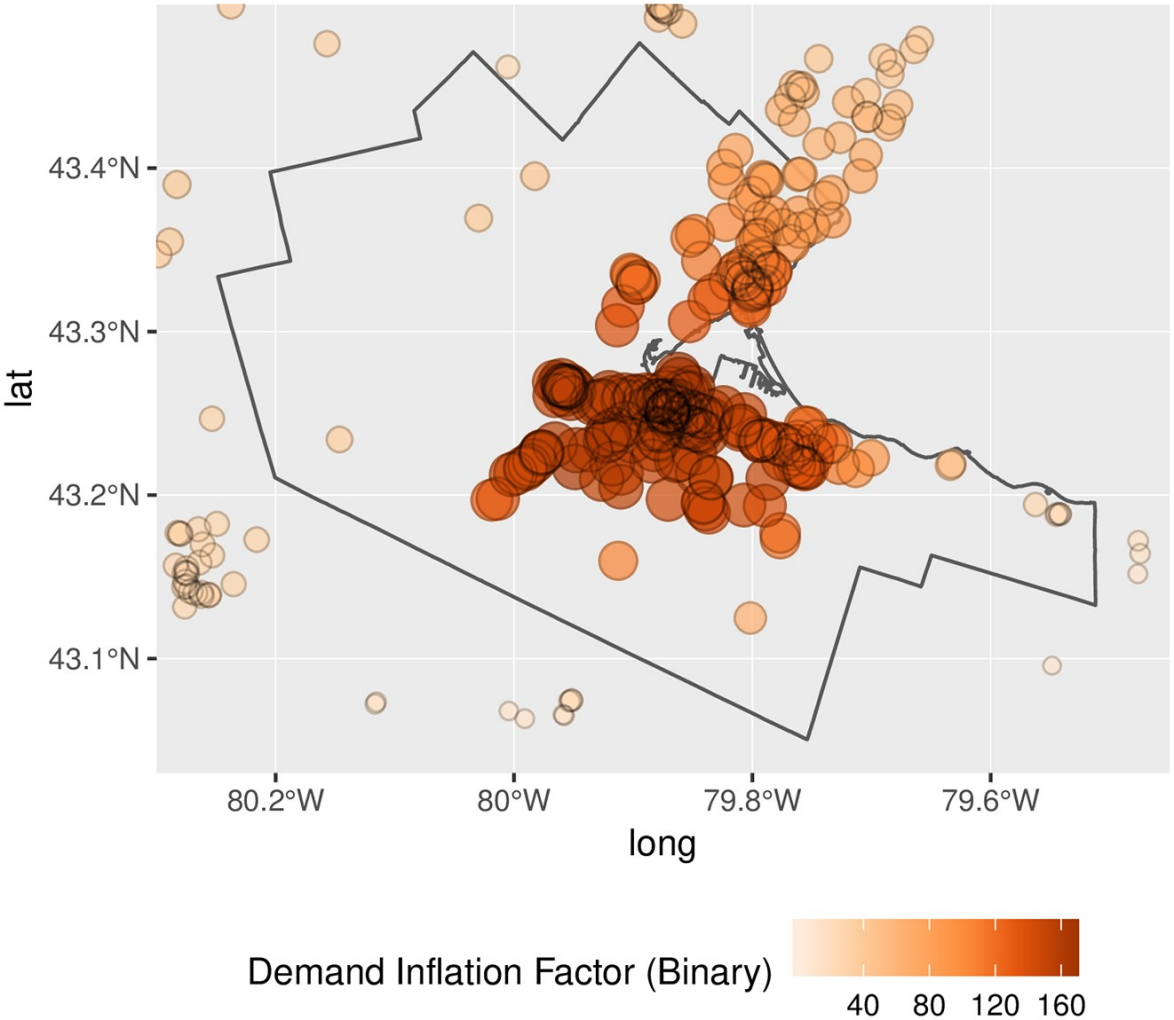
Demand inflation, binary impedance function.

**Fig 13:**
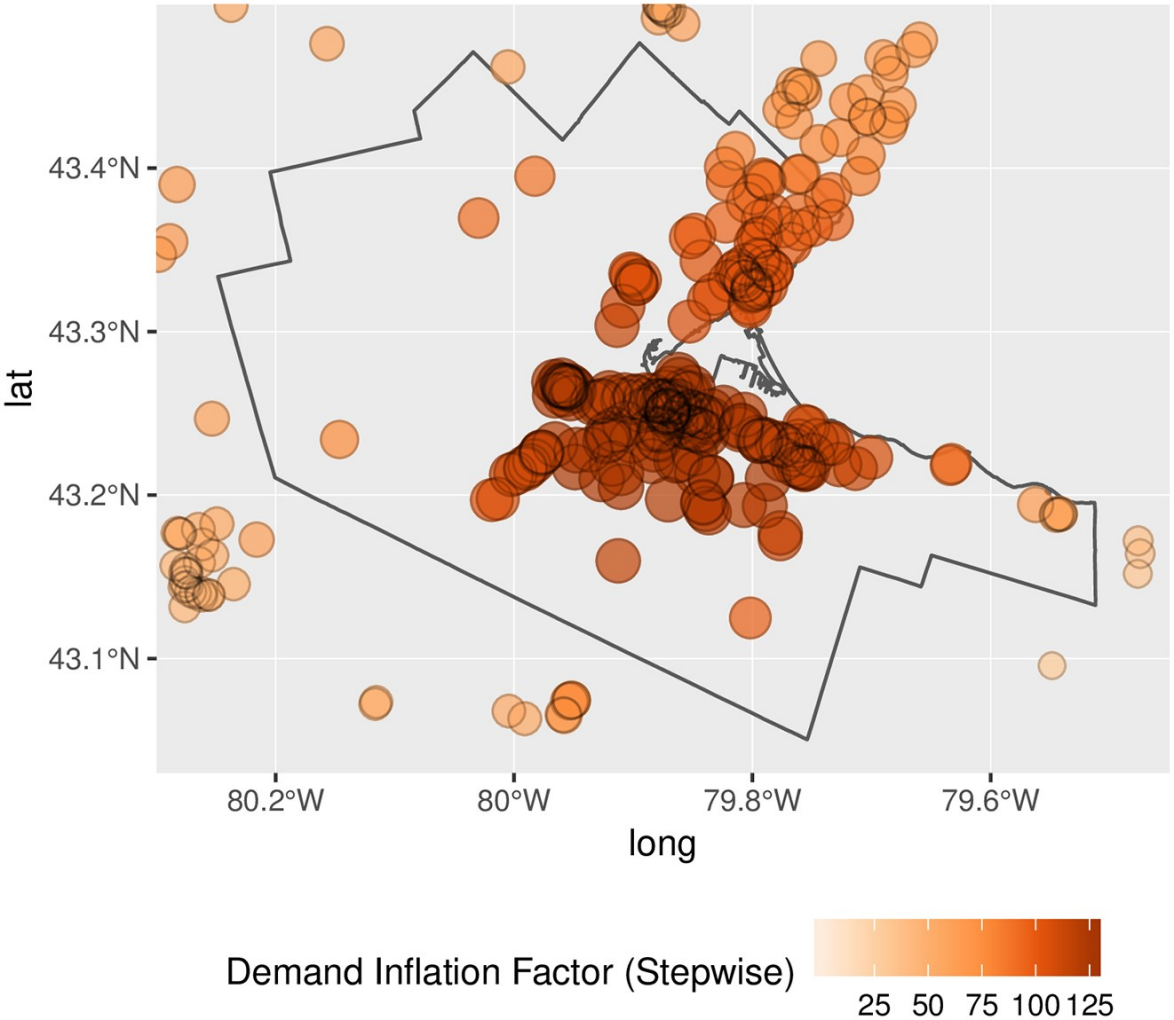
Demand inflation, stepwise impedance function.

Another important issue is that spatial distribution of inflation of demand and level of service. If inflation happened in a uniform way, the upward bias in the estimates could to some extent be ignored, as long as relative differences by location remained relatively constant. Unfortunately, as seen in Figs 12 and 13, demand inflation is far from uniform. In fact, inflation of demand tends to happen, as per our earlier conjecture, in areas with higher population density. Inflation factors are also substantially higher when the binary impedance function is used. Since this function lacks a gradual distance-decay mechanism, it is more generous in terms of counting population serviced. Notice the magnitude of the inflation factors: since the inflation of demand depends on the number of overlapping catchment areas, a factor of 160, for instance, would suggest that a clinic is expected to *simultaneously* serve approximately that number of DAs in the conventional 2SFCA method, and a proportionally similar number in the conventional E2SFCA method.

**Fig 14:**
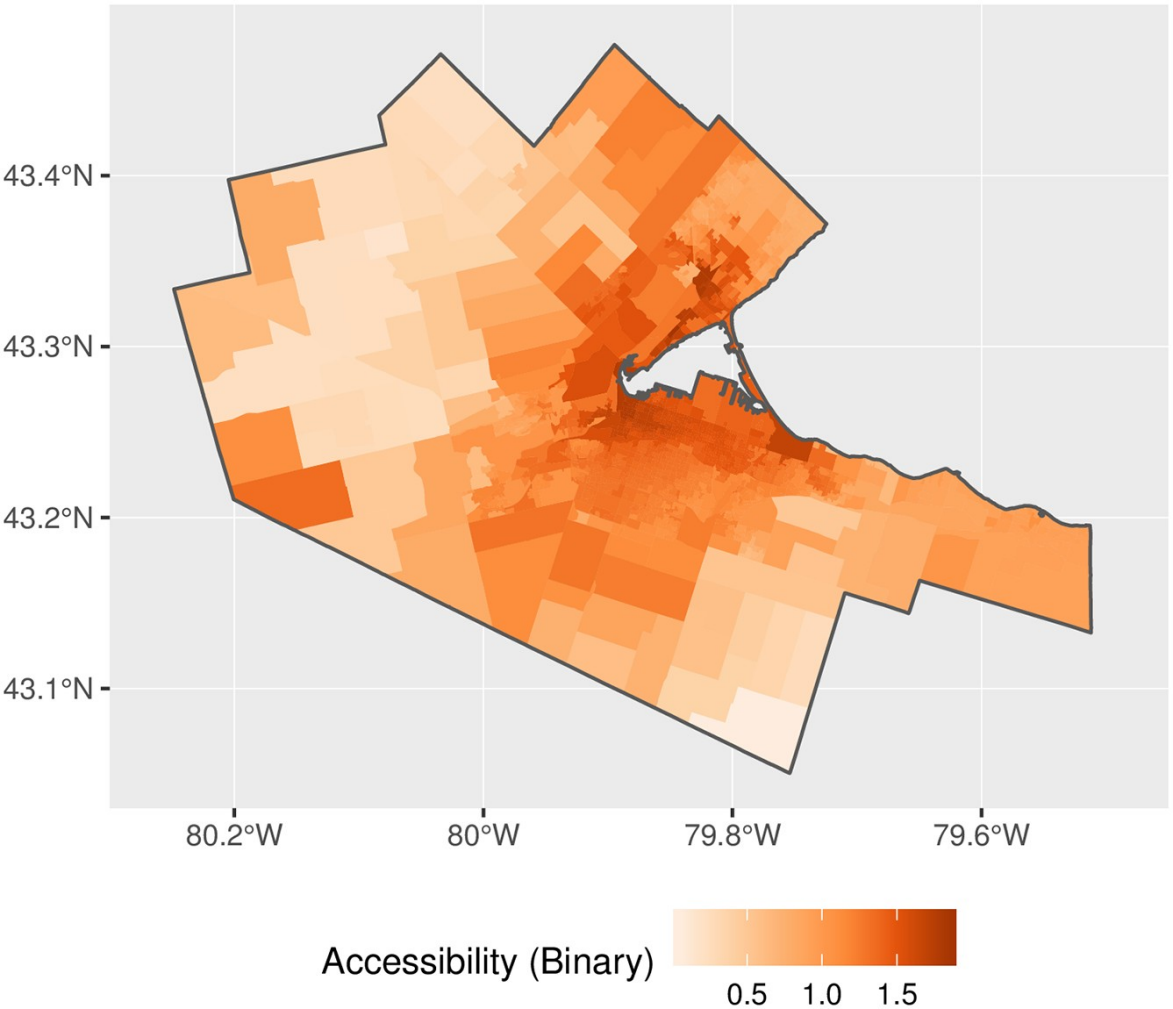
Accessibility, binary impedance function.

**Fig 15:**
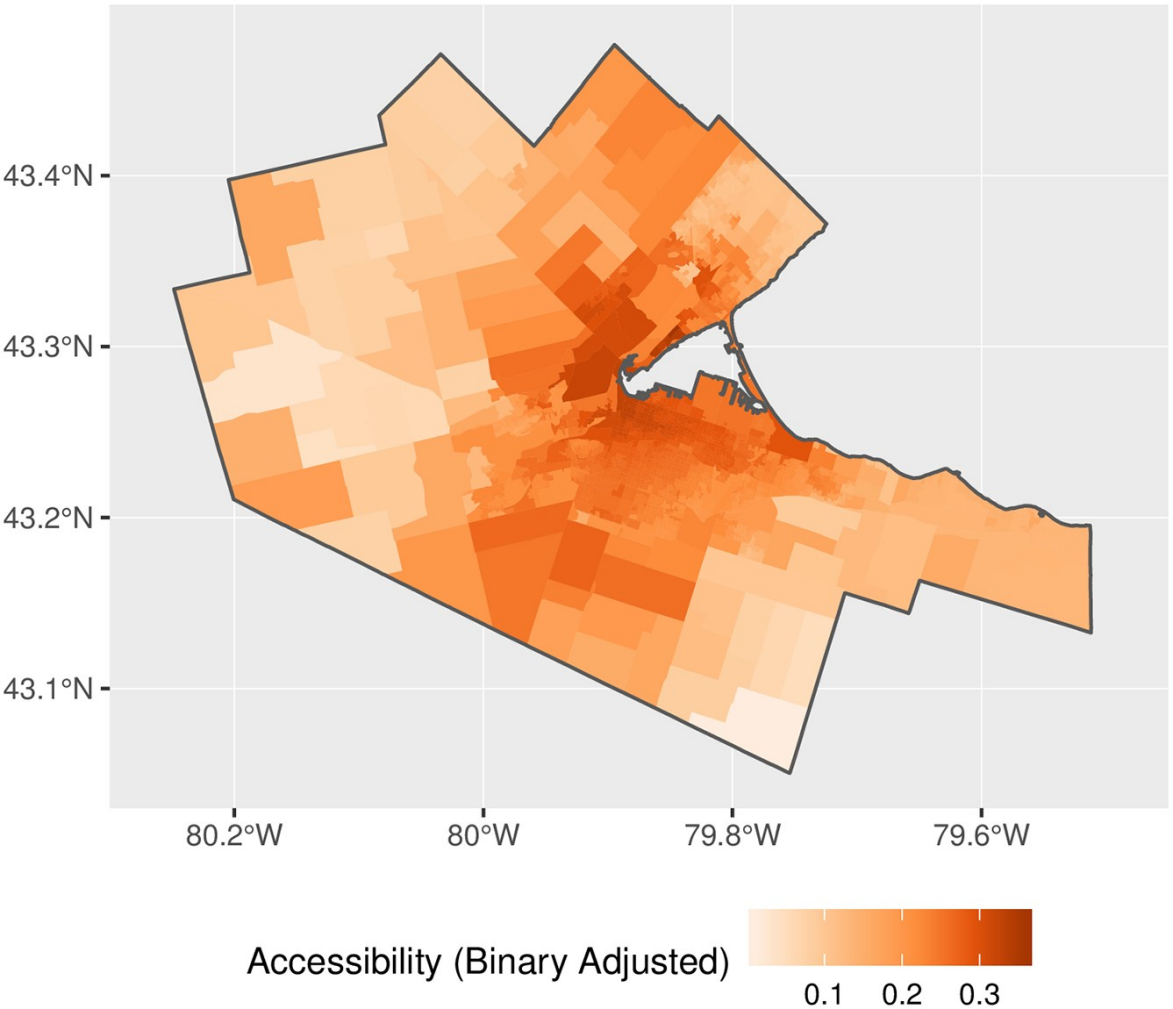
Accessibility, adjusted binary impedance function.

**Fig 16:**
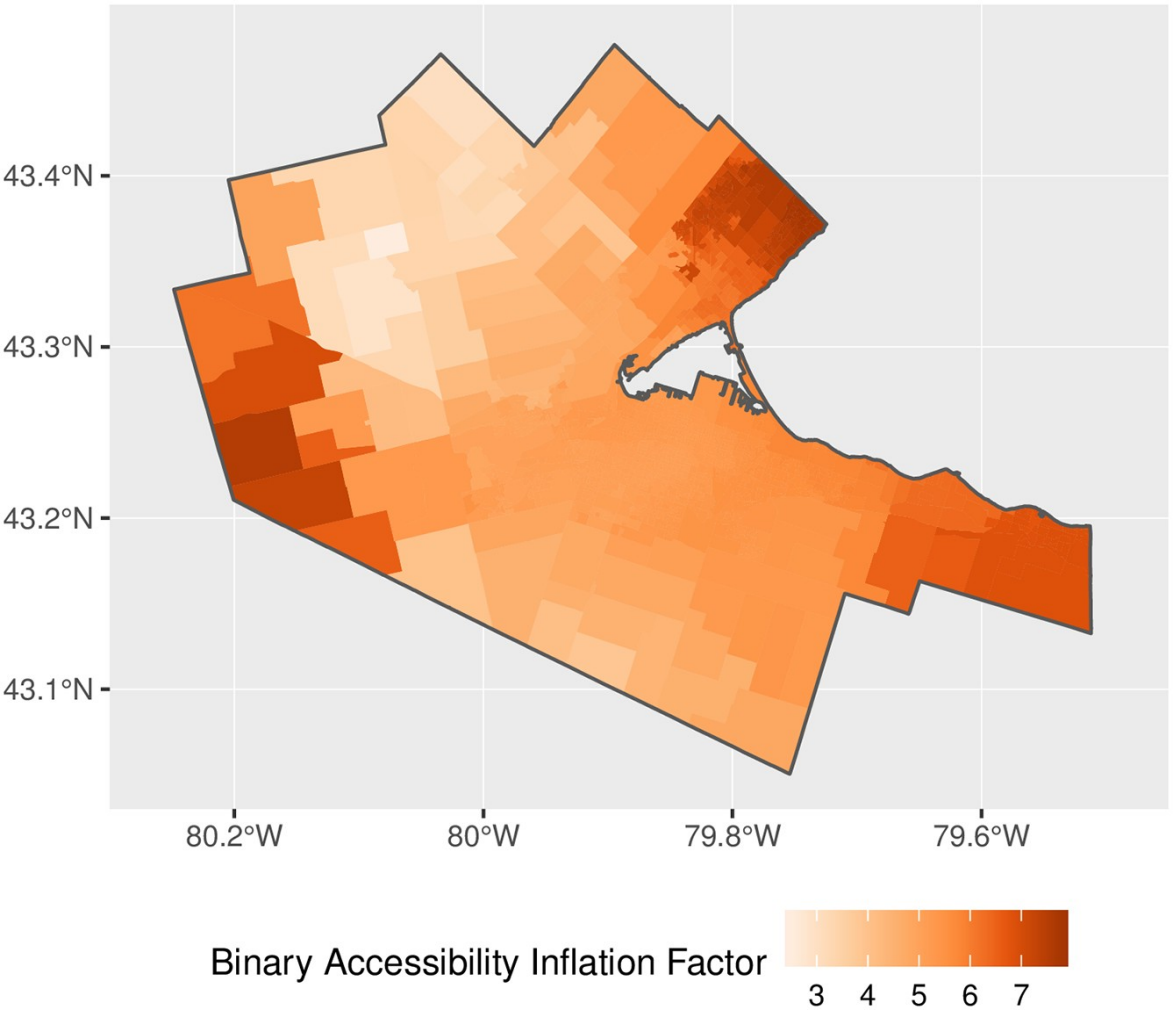
Accessibility inflation factor, binary impedance function.

The map of accessibility for the implementation of 2SFCA is shown in Fig 14 and with the adjusted weights for proportional allocation in Fig 15. The general patterns observed in the figures are as expected, with higher accessibility in denser, better connected parts of the region. Relatively high accessibility in the north and west of the CMA is due to proximity to other major population centers such as Oakville, Kitchener, and Waterloo. A question, however, is the degree of inflation of accessibility in the original 2SFCA? Fig 16 plots the ratio of the binary and adjusted binary accessibility measures. Here it can be seen that the unadjusted accessibility values are at least three times greater than their adjusted counterparts within the study area. This inflation, moreover, is not uniform across space, with inflation of the binary accessibility values up to 8 times greater than those from the adjusted model at the edges of the city where the 15-minute catchment areas begin to overlap with neighboring municipalites.

**Fig 17:**
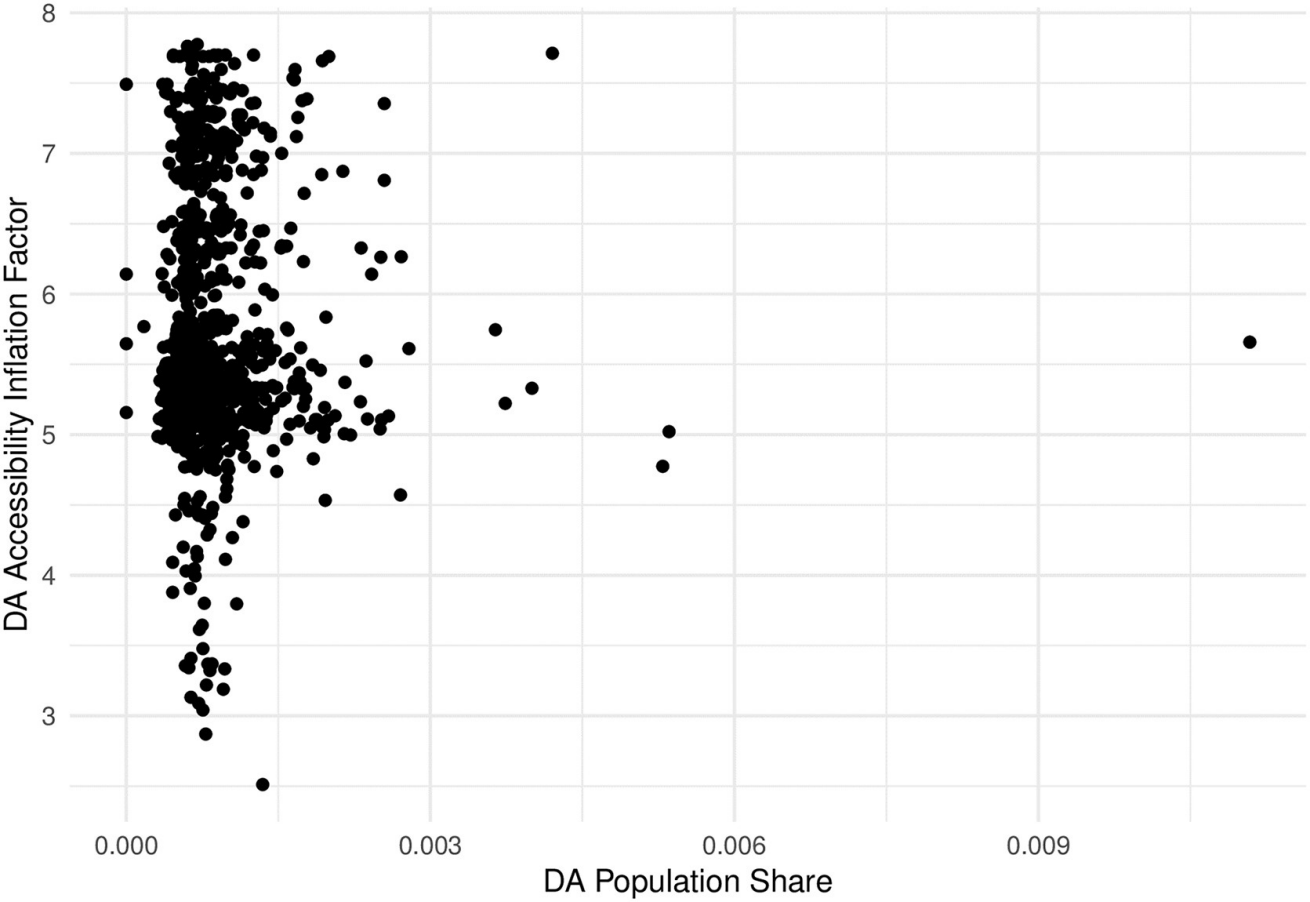
Population share and inflation factors compared.

Why is this important? As noted by various authors [3, 18], in traditional FCA methods, the sum of the population-weighted average of accessibility across all population centers is equal to the regional average provider-to-population ratio [18]. In the present case, the weighted sum of accessibility in the unadjusted binary and stepwise measures is 0.751. However, while this value is indeed identical to the regional average provider-to-population ratio, it is problematic because the share of the population correlates poorly with the pattern of inflation observed (see Fig 17). The key issue here is that accessibility is deflated by the share of the population in a DA *i*; however, the degree of inflation of demand and supply depends not only of the population DA *i*, but on the population of every DA *j* with which DA *i* interacts via overlapping catchment areas. As a consequence, deflating accessibility using population shares in previous FCA methods does not accurately offset demand and supply inflation.

**Fig 18:**
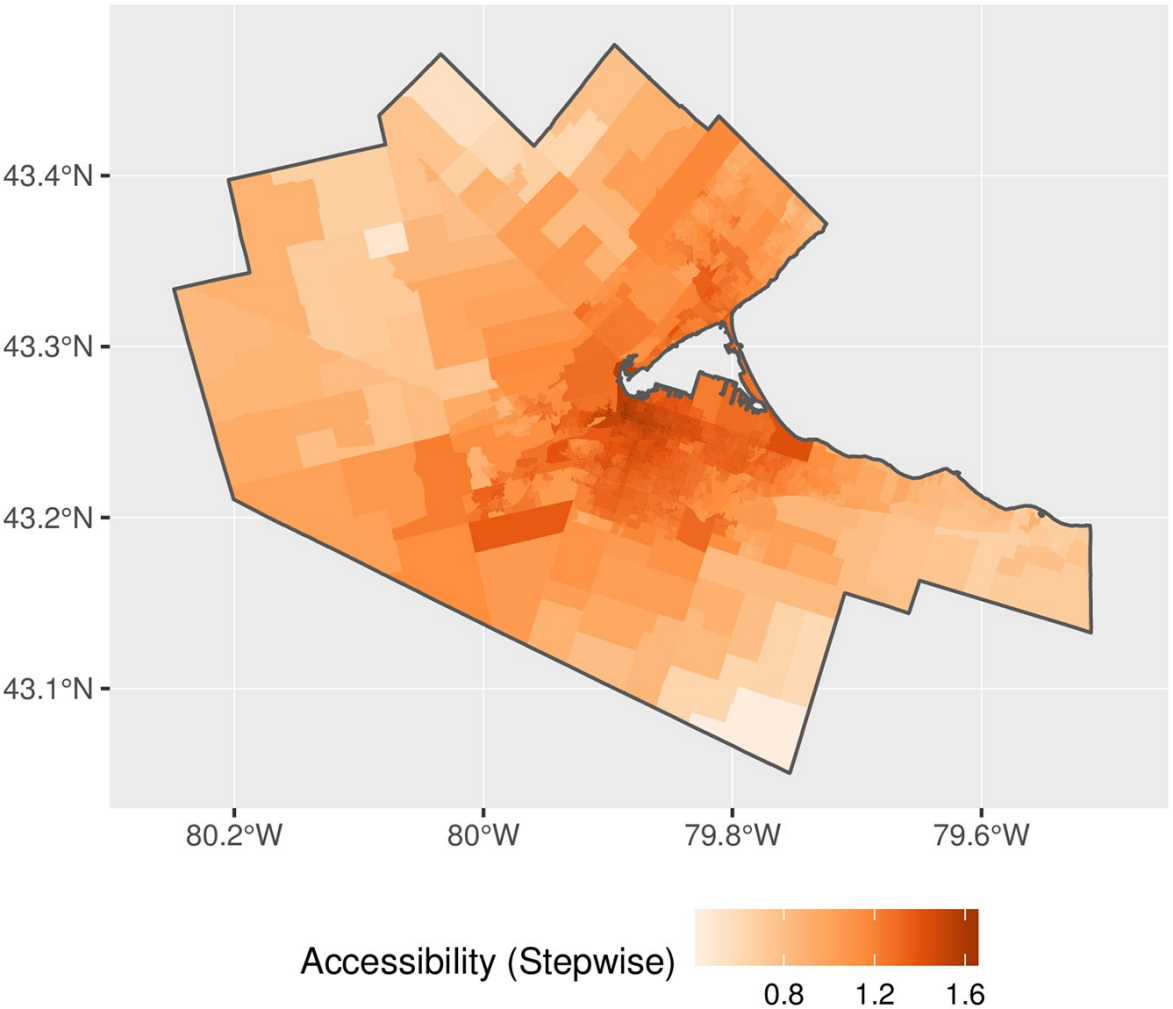
Accessibility, stepwise impedance function.

**Fig 19:**
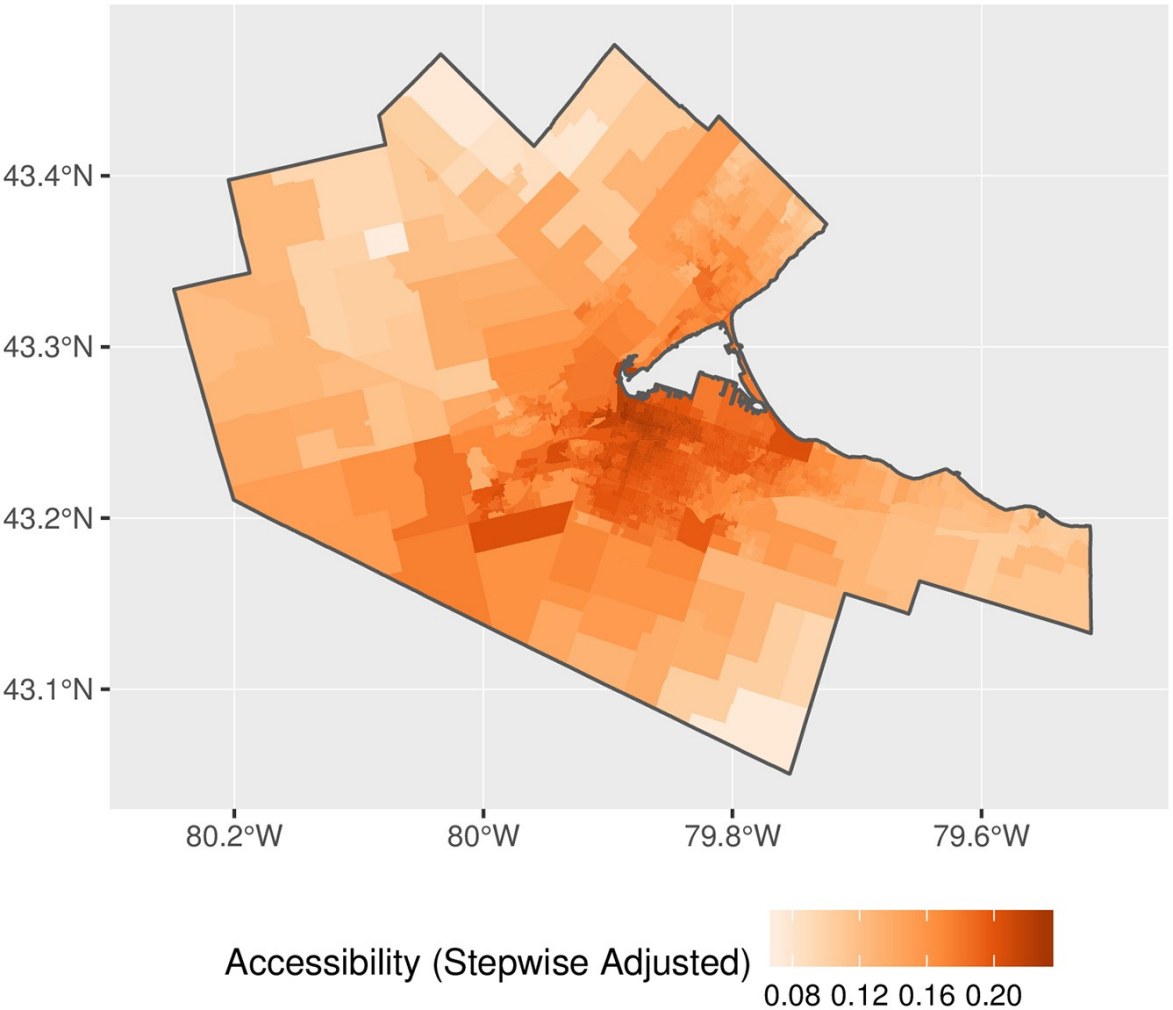
Accessibility, adjusted stepwise impedance function.

**Fig 20:**
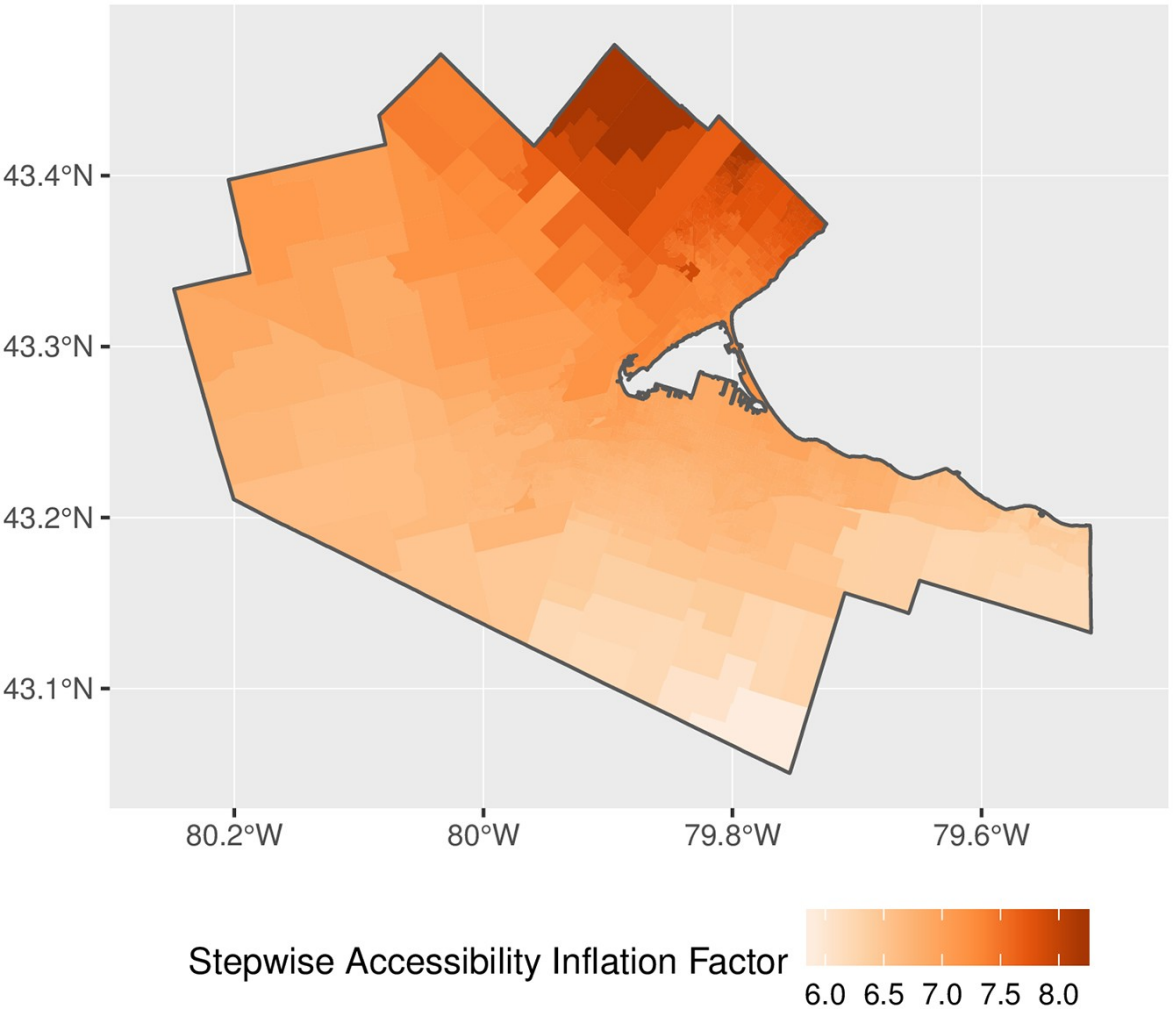
Accessibility inflation factor, stepwise impedance function.

Figs 18 and 19 present the results for the stepwise E2SFCA with and without the rectification. The results are qualitatively similar to the 2FSCA, with the expected differences (see Fig 20). The inflation factors are even more substantial, given the larger catchment areas used.

### Disparity analysis

An advantage of the use of adjusted weights for proportional allocation of demand and level of service it that, after rectifying the inflation artifact, they make it is possible to conduct accessibility disparity analysis in a very intuitive way.

For instance, an analyst interested in equity analysis could allocate the total level of service uniformly to every DA. In other words, the total level of service (which equals the sum of accessibility over the system) can be divided by the number of population centers in the system to return the Average Local Population Center PPR. The resulting mean value, call it $L_{i}^{e}$ then would be assigned to the population centers as their “equitable” share of the total level of service in the system. Next, the equitative distribution of the level of service in each population center is substracted from the estimated mean accessibility to arrive at a disparity index. When the difference between these two quantities is positive, this would indicate that a DA’s accessibility exceeds its equitable share of level of service. On the other hand, when the difference is negative, the DA’s accessibility is below its equitable share of the level of service.

This approach is reminiscent of the Spatial Access Ratio (SPAR) proposed by Wan et al. [28], which is calculated as the ratio between a population center’s accessibility and the mean accessibility across all population centers. Wan et al. [17] calculate SPAR based on the results of their 3SFCA method, by rescaling the accessibility measures to reflect the percentage difference in each population center’s accessibility relative to the mean. This measure is designed to overcome the sensitivity of existing FCA metrics to the impedance function. In contrast, the approach proposed here, enables more intuitive and interpretable results by preserving the system-wide population and level of service. In this way, a disparity index is useful to highlight the absolute difference in accessible provider-to-population ratios across population centers.

**Fig 21:**
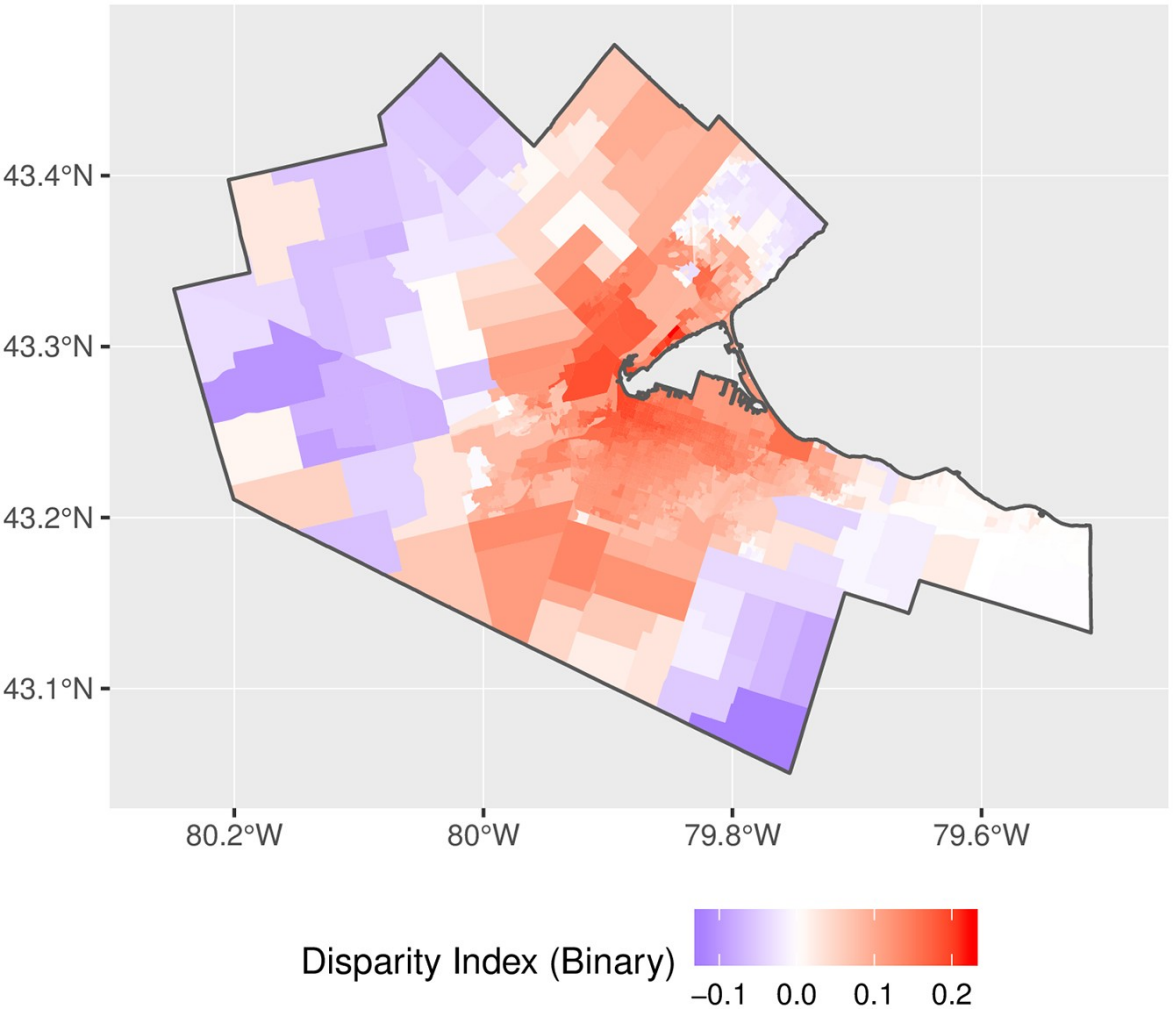
Accessibility disparities, adjusted binary impedance function.

**Fig 22:**
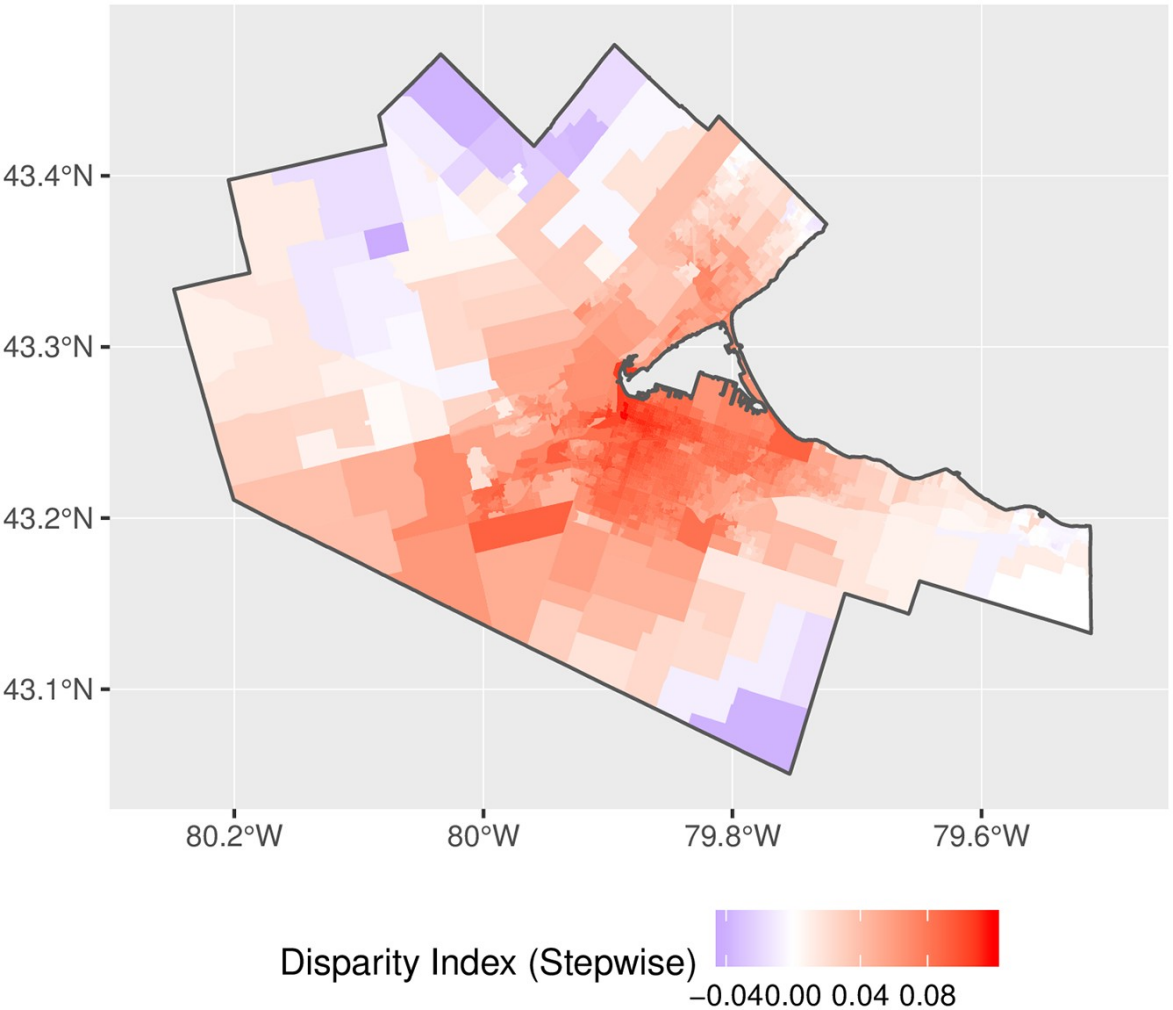
Accessibility disparities, adjusted stepwise impedance function.

Disparity maps for the adjusted binary and stepwise impedance functions are shown in Figs 21 and 22. These figures reveal the spatial distribution in disparity, with levels of access that are lower than the mean in more rural parts of the city (where travel times are longer and the distribution of physicians is more spatially disperse) compared to levels of access that are greater than the mean in the higher-density and more connected urban center.

## Conclusion

Accessibility to healthcare is an issue of continued interest in health geography. One of the most popular approaches to estimating accessibility is the 2SFCA method and its associated family of FCA models due to their simplification of more complex gravity models and their interpretation as proxies for provider-to-population ratios. These properties make FCA approaches particularly appealing for health policy. In this paper, we have argued that the overestimation of demand and level of service in FCA approaches poses a challenge to the interpretation of accessibility and the identification of spatial disparities in access, with potentially deleterious consequences for policy analysis.

The issue of overestimation of demand and level of service has been recognized before, notably by Wan et al. [17] and Delamater [18], and alternative approaches have been proposed that seek to offset or reduce the problem. Nevertheless, the present paper has shown that the inflation of demand is present in all existing FCA methods. Moreover, we also show that in some cases, demand is deflated, and detail the potential for inflation/deflation on the supply side. To overcome these issues, we draw from the fields of spatial statistics and econometrics, to incorporate row-standardized impedance weights in the calculation of demand, and column-standardized impedance weights to adjust the level of service. These adjustments ensure that allocation of demand and level of service are proportional. As a result, both the system-wide population and level of service are preserved in the estimation of accessibility.

The case study in Hamilton CMA reveals the extent of inflation in accessibility inherent in the unadjusted approaches compared to the adjusted binary and stepwise FCA methods. Furthermore, the adjustments result in local provider-to-population ratios which can be easily understood relative to the system-wide equitable level of service through the calculation of a disparity index. The applicability of these values is particularly enhanced by the use of a travel survey to inform the estimated impedance functions. Taken together, these innovations provide estimates of spatial accessibility and disparity that are robust to the regional distribution of supply and demand, as well as observed travel behaviour. By extension, these properties mean that the adjusted approach employed here can offer more rigorous recommendations for health policy.

Finally, 1) we proposed a set of slack factors to modulate the estimates of demand and/or level of supply to account for system inefficiencies; and 2) demonstrated the use of a disparity index to conduct equity analysis.

In conclussion, the research presented in this paper demonstrates how a relatively simple adjustment of the impedance weights can help to overcome the inflation/deflation issue inherent in previous FCA approaches. By incorporating these methods into the estimation of accessibility to healthcare services, future research can help to ensure that the FCA approach continues to live up to its promise as an intuitive and policy-relevant method for investigating access and disparity.

## Replicable research

This research was conducted and the paper prepared using R and RStudio, along with the following packages: tidyverse [29], knitr [30], rgdal [31], sf [32], gridExtra [33], raster [34], readr [35], kableExtra [36], ggthemes [37], ggrepel [38]. Data and source code used in this research are available at https://github.com/paezha/Demand-and-Supply-Inflation-in-Floating-Catchment-Area-FCA-Methods-.
